# Surgical patient-reported experience measures and qualitative experience studies: systematic review

**DOI:** 10.1093/bjsopen/zrae142

**Published:** 2025-02-11

**Authors:** Maram Darwish, Shabita Nandy, Simone Willis, James Coulson, Kathleen Withers, David C Bosanquet

**Affiliations:** Southeast Wales Vascular Network, University Hospital of Wales, Cardiff, UK; Health Education England, The East Midlands Deanery, Leicester, UK; Cardiff University, School of Medicine, University Hospital of Wales, Cardiff, UK; Centre for Healthcare Evaluation, Device Assessment and Research, CEDAR, Cardiff and Vale University Health Board, Cardiff, UK; Specialist Unit for Review Evidence, Cardiff University, Cardiff, UK; Cardiff University, School of Medicine, University Hospital of Wales, Cardiff, UK; Centre for Healthcare Evaluation, Device Assessment and Research, CEDAR, Cardiff and Vale University Health Board, Cardiff, UK; The Welsh Value in Health Centre, Cwm Taf University Health Board, Cardiff, UK; School of Engineering, Cardiff University, Cardiff, UK; Southeast Wales Vascular Network, University Hospital of Wales, Cardiff, UK; Department of Vascular Surgery, Aneurin Bevan University Health Board, Newport, UK

## Abstract

**Background:**

Patient-reported experience measures are tools used to gather feedback from patients about their experiences of healthcare services, which are crucial for improving the quality of care from the perspective of patients. The aim of this systematic review was to identify surgery-related patient-reported experience measures, evaluate their psychometric properties, appraise and identify recurring themes within qualitative studies on surgical care, and identify potential bias in study designs.

**Methods:**

PubMed, MEDLINE, Embase, the Cumulative Index to Nursing and Allied Health Literature database, and the Cochrane Library, along with clinical trial registries, were searched for articles on surgery-specific patient-reported experience measures and qualitative studies on patients’ experiences up to 21 September 2023. Manual coding was used for theme identification and grouping based on thematic synthesis principles. Joanna Briggs Institute tools were used for risk-of-bias assessment and a revised version of the COnsensus-based Standards for the selection of health Measurement INstruments checklist was used for appraisal of patient-reported experience measures.

**Results:**

A total of 14 studies met the inclusion criteria, identifying seven patient-reported experience measures. Key patient experience themes included communication with healthcare providers, care setting environment, overall satisfaction, pre-admission information, coordination of care, waiting time, surgical experience, post-surgery support, impact on life, and healthcare information and technology management. Internal consistency was reported adequately across all patient-reported experience measures. Other psychometric properties were questionable.

**Conclusion:**

Inadequate psychometric evaluations of some patient-reported experience measures in surgery highlight the need for rigorous validity and reliability assessments. Identification of thematic patterns emphasizes the importance of ongoing research to explore patients’ experiences in surgical contexts. Clinical staff can use this information to enhance communication, reduce waiting time, and improve the overall patient experience by addressing highlighted areas.

## Teaser

This systematic review identifies and evaluates surgery-specific patient-reported experience measures, highlighting key patient experience themes, such as communication, care coordination, and waiting time. The study underscores the need for rigorous psychometric assessments of patient-reported experience measures to enhance overall patient care in surgical contexts.

## Introduction

Patient-reported experience measures (PREMs) are tools used to gather feedback from patients about their experiences of healthcare services. Whereas patient-reported outcome measures (PROMs) focus on the outcomes of treatments, PREMs concentrate on patients’ perception of the care they received^1,2^. This includes aspects such as communication with healthcare providers, accessibility of services, and continuity of treatment^3^.

Unlike satisfaction surveys that present subjective and generic assessment of whether the care met patients’ expectations or not^4–7^, PREMs provide objective and quantifiable representation of the quality of care. Ideally, all PREMs should be validated; the reliability and validity assessments of PREM tools should confirm their accuracy and consistency in capturing relevant data^7–12^. Several PREMs have been developed and validated within the broader healthcare landscape^13–19^; however, few PREMs specifically focus on surgery.

Incorporating PREMs into clinical practice is an essential part of patient-centred care. PREMs help clinicians identify areas affecting patients’ healthcare journey and can reveal communication gaps between surgeons and patients, facilitating better compliance with management plans and recovery processes^2,20^. Insights from PREMs can help clinicians tailor care to individual patient needs, resulting in more personalized and effective treatment plans.

Qualitative studies of patients’ experiences often capture the intricacies of patients’ experiences in healthcare^21–24^. Whereas PREMs provide quantitative data on experience, qualitative feedback provides more granular data on patients’ lived experiences.

The aim of this systematic review was to undertake a comprehensive evaluation of the validated PREMs used in surgery and qualitative studies exploring the experiences of surgical patients.

## Methods

This study followed the PRISMA statement^25^; PRISMA (*Supplementary S1*) and PRISMA-S (*Supplementary S2*) checklists were completed. The review protocol was prospectively registered in PROSPERO, the international prospective register of systematic reviews (CRD42023479711)^26^. See *Supplementary S3* for deviations from the original protocol.

### Study objectives

The study objectives were: to identify PREMs designed for assessing patients’ experiences in surgery and surgical sub-specialties; to identify domains and items utilized in each PREM; to identify themes that emerged from qualitative research assessing surgical patients’ experiences; and to assess the methodological quality of the PREMs.

### Eligibility criteria

Inclusion criteria were: quantitative studies that included and/or validated PREMs within the domain of surgery, as defined by the Royal College of Surgeons of England^27^, including general surgery, vascular surgery, cardiothoracic surgery, neurosurgery, oral and maxillofacial surgery, otolaryngology, plastic surgery, trauma and orthopaedic surgery, and urology; qualitative studies that reported on patients’ perioperative experiences; participants aged 18 years or older; and studies written in English.

Exclusion criteria were: studies that focused on other specialties; studies that used PREMs where the psychometric assessment indicated insufficient validity and reliability; and studies that measured patients’ expectations or PROMs, rather than patients’ experiences.

### Information sources

A search strategy was devised to identify pertinent studies, encompassing electronic databases, including PubMed, MEDLINE (Ovid), Embase (Ovid), the Cumulative Index to Nursing and Allied Health Literature (CINAHL) database (via EBSCO), and the Cochrane Library. The search strategy was developed in MEDLINE (Ovid) and translated to the other databases. Search terms were developed in consultation with a university librarian. Additionally, clinical trial registries, including ClinicalTrials.gov, the European Union Clinical Trials Register, and the ISRCTN registry, were searched using relevant terms. The search strategy included controlled vocabulary and free-text terms encompassing the definition of PREMs, surgery, and surgical sub-specialties^27^. Due to resource constraints, searches were limited to studies written in English. The search strategy was reviewed by research librarians using the Peer Review of Electronic Search Strategies (PRESS) checklist^28^. Searches were conducted from database inception to 21 September 2023 (*Supplementary S4*). Backward citation searching was undertaken for the included papers.

Endnote (version 20) was used for reference management and removal of duplicates. Two reviewers (M.D. and S.N.) independently screened the titles and abstracts of articles. Where disagreements occurred, articles were included for full-text review. M.D. and S.N. independently conducted full-text screening; disagreements were resolved through discussion.

### Risk of bias

The methodological quality of the included studies was assessed using the Joanna Briggs Institute (JBI) critical appraisal tools^29^. The JBI tools accommodate quantitative^30^ and qualitative^28^ study designs. For this review, the JBI critical appraisal checklist was used for: case–control studies (*Supplementary S5*); cross-sectional studies (*Supplementary S6*); and qualitative studies (*Supplementary S7*).

For the quality appraisal of PREMs, the revised COnsensus-based Standards for the selection of health Measurement INstruments (COSMIN) checklist^31^ was used. M.D. and S.N. conducted the quality assessment independently in duplicate. Disagreements were resolved through discussion.

### Data extraction

Data extraction was performed independently in duplicate by M.D. and S.N. Discrepancies were resolved through discussion. A standardized form was used for data extraction. The following information was extracted from each quantitative study: author(s), year of publication, country of origin, sample size, study population, PREM used, mode of administration, and PREM characteristics. For the qualitative studies, the data extracted were: author(s), year of publication, country of origin, sample size, study population, qualitative method used, and main themes identified.

### Surgical patients’ experiences—theme development

Aligning with similar methodologies utilized in previous literature on PREMs^32^, the aim was to identify and categorize emerging themes from PREMs and qualitative studies assessing patients’ experiences within surgical contexts. One reviewer (M.D.) identified and categorized thematic patterns emerging from the reviewed studies using manual coding and guided by thematic synthesis principles^33^. The themes were reviewed and ratified by the co-authors.

## Results

A total of 5214 records were identified from database searches and registers, 7 of which were removed during de-duplication. The remaining 5207 records underwent title and abstract screening, resulting in 5179 records being excluded. Thus, a total of 28 full-text articles were reviewed and 16 of these articles were excluded. Details of excluded studies can be found in *Supplementary S8*. A total of 77 articles were identified through backward citation searching and 2 of these articles were included in the review. Thus, a total of 14 articles met the inclusion criteria (*Supplementary S9*).

### Included studies

The characteristics of the included studies are summarized in *Table 1* (quantitative studies) and *Table 2* (qualitative studies). All studies were published after 2010. Sample sizes ranged from 10 to 60 526 and resulted in a total of 73 796 participants across the included studies. Of the studies, four were conducted in the USA^12,34–36^, three were conducted in the UK^37–39^, two were conducted in Sweden^40,41^, one was conducted in Denmark^11^, one was conducted in Turkey^10^, one was conducted in the Netherlands^42^, one was conducted in Spain^43^, and one was conducted in Ireland^44^. Of the studies, two were conducted among colorectal surgery patients^35,41^, two were conducted among orthopaedic surgery patients^11,40^, two were conducted among breast surgery patients^34,44^, two were conducted among general surgery patients^10,36^, one was conducted among emergency abdominal surgery patients^37^, one was conducted among gastrointestinal cancer surgery patients^12^, one was conducted among plastic and reconstructive surgery patients^42^, one was conducted among elective day-case surgery patients^39^, one was conducted among urology patients^38^, and one was conducted among transplant surgery patients^43^. A total of nine studies used quantitative study designs to report on patients’ experiences or the psychometric properties of a validated PREM^10–12,34–37,39,42^. With the exception of one study^34^ that used a specialty-specific PREM (the Press Ganey Ambulatory Surgery Survey (PGASS) to assess the ambulatory surgery service), all studies used generic PREMs to assess surgical patients’ experiences. A total of five studies examined patients’ experiences through qualitative research design^38,40,41,43,44^. The qualitative studies used semi-structured interviews^40,41,44^, focus group sessions^38^, or both^43^. Apart from one study^43^ that recruited patients and healthcare providers (HCPs) for data collection, all of the qualitative studies only recruited patients^38,40,41,44^.

**Table 1:** Characteristics of quantitative studies reviewed

Study	Country	Sample size	Study population (surgical condition studied)	Instrument used (PREM)	Number of items/domains	Domains measured	Mode of administration
Wick *et al*.^35^, 2015	USA	640	Colorectal surgery	HCAHPS	27/9	Nurse communication Doctor communication Staff responsiveness Pain management Communication about medications Discharge information Cleanliness of the hospital environment Quietness of the hospital environment Transition of care	PREM data were obtained from the HCAHPS survey, publicly available on the CMS Hospital Compare website as hospital-level scores
Jones *et al*.^37^, 2017	UK	68	Emergency abdominal surgery	NHS adult inpatient survey (GIS)	50/7	Admission Ward environment Patient–staff interaction (doctors) Patient–staff interaction (nurses) Information and involvement with treatment Discharge Overall experience	Survey was administered either over the phone shortly after discharge or in person at the time of discharge
Liu *et al*.^12^, 2019	USA	60 526	Gastrointestinal cancer surgery	HCAHPS	27/9	Nurse communication Doctor communication Staff responsiveness Pain management Communication about medications Discharge information Cleanliness of the hospital environment Quietness of the hospital environment Transition of care	PREM data were obtained from the HCAHPS survey, publicly available on the CMS Hospital Compare website as hospital-level scores
Hertel-Joergensen *et al*.^11^, 2018	Denmark	215	Orthopaedic surgery	GPNCS	34/7	Physical care Giving information Support Respect Personnel characteristics Environment Nursing process	The patients completed the electronic questionnaire using a tablet computer on the first day after surgery or after discharge from ICU
Donmez and Ozbayır^10^, 2011	Turkey	346	General surgery	GPNCS	34/7	Physical care Giving information Support Respect Personnel characteristics Environment Nursing process	The patients completed the questionnaire in paper form distributed by the research team during their inpatient stay
Murphy *et al*.^34^, 2019	USA	270	Breast surgery	PGASS	32/7	Registration Nursing Surgeon Facility Personal issues Patient safety Overall assessment	The records were retrieved retrospectively regarding patients who underwent a breast operation from July 2015 to December 2016 and completed a survey within 2 weeks of discharge
Poelstra *et al*.^42^, 2018	Netherlands	836	Plastic, reconstructive, and hand surgery	Nationally developed PREM	25/6	Physician communication and competence Perioperative care Postoperative care General information Treatment information Quality of facilities	Patients who underwent the surgery were invited to complete a PREM questionnaire 3 months afterwards; two reminders were mailed to non-responders
Schreiter *et al*.^36^, 2021	USA	437	General surgery (abdominal surgery)	Press Ganey Survey	24/6	Access Moving through the visit Nurse assistant Care provider Personal issues Overall assessment	Filled in forms retrieved from patients’ medical records
Black *et al*.^39^, 2014	UK	10 383	Elective surgery (hip replacement, knee replacement, and hernia repair)	PPE-15	32/8	Information and education Coordination of care Physical comfort Emotional support Respect for patients’ preferences Involvement of family and friends Continuity and transition Overall impression	PREM questionnaires were mailed 6 weeks after surgery to all patients who had completed a preoperative PROM questionnaire

PREM, patient-reported experience measure; HCAHPS, Hospital Consumer Assessment of Healthcare Providers and Systems; CMS, The Centers for Medicare and Medicaid Services; GIS, General Inpatient Survey; GPNCS, Good Perioperative Nursing Care Scale; PGASS, Press Ganey Ambulatory Surgery Survey; PPE-15, a 15-item short form of the Picker Patient Experience Questionnaire; PROM, patient-reported outcome measure.

**Table 2:** Characteristics of qualitative studies reviewed

Study	Country	Study participants	Sample size	Study population (surgical condition studied)	Qualitative method	Main themes
Ventura-Aguiar *et al*.^43^, 2022	Spain	Semi-structured interviews	13 healthcare professionals 12 SPKT patients	Transplant surgery	Focus groups and semi-structured interviews	Contact and communication Information Waiting time Impact on patients’ everyday life Information received before consultation with the transplant unit Information received at the transplant unit and before the surgery Areas of improvement Post-surgery requirements
Harrison *et al*.^38^, 2023	UK	Patients who had experience of kidney cancer follow-up care after surgery	14 participants	Urology (kidney cancer surgery)	Focus groups	Feelings of abandonment Uncertainty about the plan Anxiety about appointments Variation in care A need for information A need for emotional support
Brennan *et al*.^44^, 2020	Ireland	Breast cancer survivors	10 participants	Breast surgery	Semi-structured interviews	Rehabilitation Experience with digital technology
Arvidsson *et al*.^40^, 2023	Sweden	Patients who underwent DRF surgery and were discharged home	10 participants	Orthopaedic surgery	Semi-structured interviews	The video call—new, but surprisingly simple The video call—the patient’s choice
Lithner *et al*.^41^, 2015	Sweden	Patients who underwent colorectal cancer surgery and were discharged home	16 participants	Colorectal surgery	Semi-structured interviews	Trying to regain control in life by using information: using information to make daily life work; wanting to partake in the information; and needing information to manage the worries and make the disease comprehensible

SPKT, simultaneous pancreas-kidney transplant; DRF, distal radius fracture.

### Characteristics and performance of patient-reported experience measures

In total, seven PREMs were evaluated across the reviewed studies: the Hospital Consumer Assessment of Healthcare Providers and Systems (HCAHPS)^45^ was utilized by Wick *et al*.^35^ and Liu *et al*.^12^; the NHS adult General Inpatient Survey (GIS)^46^ was employed by Jones *et al*.^37^; Hertel-Joergensen *et al*.^11^ and Donmez and Ozbayır^10^ used a version of the Good Perioperative Nursing Care Scale (GPNCS)^47^ that was translated to Danish and Turkish respectively; Press Ganey Patient Satisfaction Surveys^48^ were used by Murphy *et al*.^34^ (PGASS) and Schreiter *et al*.^36^ (Press Ganey Patient Satisfaction Survey); a non-specified Dutch national PREM was utilized by Poelstra *et al*.^42^; and, lastly, a 15-item short form of the Picker Patient Experience Questionnaire (PPE-15)^47^ was used by Black *et al*.^39^ All PREMs were self-completed by patients. The time interval for survey collection from discharge to completion varied from 2 to 12 weeks post-discharge. Only two studies reported survey collection within an inpatient setting^10,11^. Of the studies, four reported aspects of psychometric testing^10,11,39,42^, two of which reported on validity^10,11^, three of which reported on internal consistency^10,11,39^, two of which reported on reliability^10,11^, and three of which reported on responsiveness^10,39,42^ (*Table 3*). The PREMs that were used consisted of 24 to 50 items (across 6 to 9 domains). Domain contents and names varied; however, the essential characteristics identified by the Patient-Reported Indicator Survey (PaRIS) initiative^49^ were encompassed within the domains of these PREMs, suggesting a level of alignment with the international fundamental patient-reported indicators.

**Table 3:** Tool assessment using the COnsensus-based Standards for the selection of health Measurement INstruments checklist

PREM	Study	Structural validity	Internal consistency	Reliability	Measurement error	Hypotheses testing	Cross-cultural validity	Criterion validity	Responsiveness
HCAHPS	Wick *et al*.^35^, 2015	Indeterminate	Sufficient	Indeterminate	Insufficient	Indeterminate	Indeterminate	Indeterminate	Indeterminate
	Liu *et al*.^12^, 2019	Indeterminate	Sufficient	Indeterminate	Insufficient	Indeterminate	Indeterminate	Indeterminate	Indeterminate
NHS adult inpatient survey (GIS)	Jones *et al*.^37^, 2017	Indeterminate	Sufficient	Indeterminate	Insufficient	Indeterminate	Indeterminate	Indeterminate	Indeterminate
GPNCS	Hertel-Joergensen *et al*.^11^, 2018	Sufficient	Sufficient	Sufficient	Indeterminate	Sufficient	Sufficient	Indeterminate	Indeterminate
	Donmez and Ozbayır^10^, 2011	Sufficient	Sufficient	Sufficient	Indeterminate	Sufficient	Sufficient	Indeterminate	Sufficient
PGASS	Murphy *et al*.^34^, 2019	Indeterminate	Sufficient	Indeterminate	Insufficient	Indeterminate	Indeterminate	Indeterminate	Indeterminate
Nationally developed PREM	Poelstra *et al*.^42^, 2018	Indeterminate	Sufficient	Indeterminate	Indeterminate	Sufficient	Indeterminate	Indeterminate	Sufficient
Press Ganey Survey	Schreiter *et al*.^36^, 2021	Indeterminate	Sufficient	Indeterminate	Insufficient	Indeterminate	Sufficient	Indeterminate	Indeterminate
PPE-15	Black *et al*.^39^, 2014	Indeterminate	Sufficient	Indeterminate	Insufficient	Sufficient	Indeterminate	Indeterminate	Sufficient

The entries ‘Indeterminate’, ‘Sufficient’, and ‘Insufficient’ are in accordance with the predefined criteria of the COnsensus-based Standards for the selection of health Measurement INstruments checklist. PREM, patient-reported experience measure; HCAHPS, Hospital Consumer Assessment of Healthcare Providers and Systems; GIS, General Inpatient Survey; GPNCS, Good Perioperative Nursing Care Scale; PGASS, Press Ganey Ambulatory Surgery Survey; PPE-15, a 15-item short form of the Picker Patient Experience Questionnaire.

### Themes identified

**Fig. 1:**
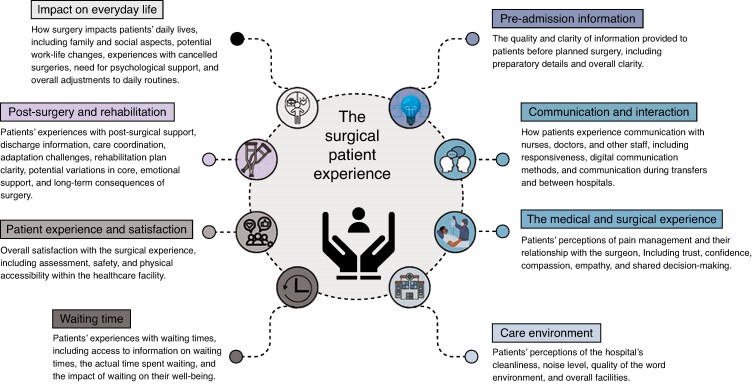
Surgical patient experience themes

Themes identified from quantitative and qualitative studies were grouped into eight primary categories that encompassed the spectrum of surgical patients’ experiences: communication and interaction; care environment; patient experience and satisfaction; waiting time; pre-admission information; post-surgery and rehabilitation; impact on everyday life; and the medical and surgical experience. The themes and subthemes are further detailed in *Fig. 1*.

### Methodological quality of the studies and the patient-reported experience measures

The included studies were rated as having good methodological quality based on the JBI tools (*Supplementary S10*). Tool assessment using the eight items of the COSMIN checklist^31^ is shown in *Table 3* and can be summarized as follows: structural validity was indeterminate for all tools, except for the translated versions of the GPNCS—in both the Turkish version of the GPNCS^10^ and the Danish version of the GPNCS^11^, structural validity was confirmed to be excellent using Confirmatory Factor Analysis (CFA) (*Supplementary S11*); internal consistency was sufficient for all tools, unanimously tested using Cronbach’s α threshold of greater than or equal to 0.70 for each unidimensional scale or subscale, mostly in previous research; reliability was indeterminate for all tools, except for the translated versions of the GPNCS^10,11^—both of the studies used Cronbach’s α coefficient and their results confirmed that both scales have good internal consistency and are reliable scales (*Supplementary S11*); measurement error was insufficient for all tools, except for the translated versions of the GPNCS^10,11^ and the Dutch national PREM^42^, for which it was deemed indeterminate; hypothesis testing was indeterminate for all tools, except for the translated versions of the GPNCS^10,11^ and the Dutch national PREM^42^, for which it was deemed sufficient; cross-cultural validity was indeterminate for all tools, except for the translated versions of the GPNCS^10,11^ and the Press Ganey Survey^36^, for which it was deemed sufficient; criterion validity was indeterminate for all tools across all studies; and, finally, responsiveness was reported according to the COSMIN checklist in only three studies^10,39,42^ with evidence provided suggesting that the GPNCS^10^, PPE-15^39^, and the Dutch national PREM^42^ have a good level of stability over time.

## Discussion

This systematic review has identified 14 studies that assessed surgical patients’ experiences and seven PREM tools that were used. All studies were published after 2010 and only three studies^10,11,39^ reported some form of additional psychometric testing. Except for one study^34^ that used a specialty-specific PREM, all included studies used generic PREMs to assess surgical patients’ experiences. In total, eight themes were extracted and consolidated to help clinicians, researchers, and policymakers to understand patients’ perspectives of their surgical journey.

The lack of a specific surgical PREM identified in the available literature and surgical care practice is noteworthy, especially given the increasing evidence supporting specialty-specific PREMs over generic ones^50–52^. Specialty-specific PREMs tailored to specific patient populations yield more accurate and meaningful data regarding patients’ experiences within the specific specialty^50–52^. Using a specialty-specific PREM facilitates a comprehensive understanding of patients’ needs, recovery experiences, and the unique challenges associated with different healthcare contexts. Implementing specialty-specific PREMs improves patient–provider communication, enhances quality of care, and facilitates targeted interventions that address the specific needs of the patient population^42,52–54^.

Incorporating specialty-specific PREMs allows for personalized feedback that serves as a motivating factor for surgeons to align their clinical practices with patient-centred care, fostering a sense of empathy and understanding for the individual experiences of their patients. The data derived from specialty-specific PREMs can support surgeons to drive quality improvement initiatives to meet the needs of their patient population.

The present analysis has identified themes that significantly impact the journeys of surgical patients (before and after surgery), including patients with different surgical pathologies, at different time points in their surgical journeys, and across different surgical settings and healthcare systems. The data support the development, validation, and improvement of surgical specialty-specific PREMs, ensuring that they are relevant and meaningful in healthcare quality assessment and improvement efforts.

To place the findings of the present study within a broader context, they were compared with key themes from previous literature across medical specialties. An Australian review of PREMs for emergency care service provision found similar themes of communication, decision-making, and care environment^32^. However, studies of PREMs developed in low- and middle-income countries highlighted issues with resources, healthcare infrastructure, confidentiality, and technical capacity^55,56^, which were less prominent in the findings of the present study, showing socio-economic impacts on patient experiences^57–60^. Also, PREMs assessing emergency care services highlighted issues related to immediate access and privacy^32^, differing from the long-term patient–provider relationships in surgical care. Mental health service PREMs prioritize emotional support and personalized care, as demonstrated in the findings of the present study, but less so in surgical contexts^16,61^. The present study highlights the importance of clear communication and identifies coordination of care and pain management as more prominent issues compared with some non-surgical specialties^4,62^.

When collecting PREM data, the choice of recall interval (that is the specific time frame for which participants are asked to recall past experiences) is a critical part of study design; a recall interval that is too long can introduce measurement errors, potentially obscuring patients’ experience highlights^63^. While no single recall interval is optimal for all measures^64^, to reduce recall bias, PREMs should, ideally, be administered as close to patient discharge as possible. Deploying PREMs within a few days post-discharge captures more vivid and accurate patient recollections, as supported by studies showing a decline in recall memory accuracy over time^63–66^. Also, utilizing a combination of different methods for PREM collection, such as electronic surveys, telephone interviews, and mailed questionnaires, can improve response rates and data quality^67^.

The included studies were of good methodological quality that effectively addressed the authors’ research question. Conversely, quality appraisal of PREM performance demonstrated a limited level of information on construct validity, reliability, and responsiveness throughout the measures, except for two studies^10,11^. It is reasonable to hypothesize that these two studies were more robust in their psychometric testing and reporting because the main objective of these studies was to validate a translated version of an existing PREM. However, it is vital that studies assessing patients’ experiences should report whether the PREM used has undergone rigorous testing for validity and reliability, as this directly influences the instrument’s ability to accurately capture patient-reported healthcare experiences. The robustness of study design when reporting PREM results or assessing PREM validity and reliability is vital to ensure that the results are a dependable representation of the instrument’s capability to reflect patients’ experiences^31^.

Overall, there were significant variations in the psychometric properties of the utilized PREMs, with numerous weaknesses identified in most tools. Whereas internal consistency was reported as sufficient across the tools, other psychometric properties were reported as insufficient and/or indeterminate to varying degrees. These findings might be due to lack of adequate psychometric testing or lack of clear information regarding psychometric testing in the published text. Also, some of the psychometric testing of the tools used may be reported outside of the peer-reviewed study, which the COSMIN checklist guidance does not account for, and this might have led to an under-representation of all testing undertaken for these measures. This highlights the need for careful consideration of the specific psychometric properties of each instrument when interpreting and comparing the results of studies including PREMs. Further validation efforts are warranted to enhance the robustness of some of the PREMs used in surgical healthcare settings. It is important to note that existing instruments lacking validation on specific criteria are not inherently flawed, but rather not properly tested. While these instruments may offer valuable insights, caution should be exercised in their use as quality assessment measures.

This systematic review represents a comprehensive effort to synthesize and report all available PREMs used in surgery. Integrating both PREMs and qualitative research findings facilitated a more robust exploration of the psychosocial, emotional, and practical dimensions of patients’ experiences before, during, and after surgical interventions. The inclusion of various surgical specialties in the review contributes to its generalizability, supporting broad applicability across different surgical settings. Lastly, this review has collectively unveiled key themes that hold implications for the development of PREMs tailored to specific surgical populations. The findings form a robust foundational framework that is crucial for comprehensively understanding and addressing the surgical patient experience.

However, there are some limitations. This review was limited to studies written in English. Also, the search strategy identified few surgery-related PREMs, which may be attributed to the specificity of the surgical population and the evolving nature of PREMs within healthcare. Some PREMs might have been inadvertently excluded due to poor reporting.

The key themes identified in this review shed light on the key priorities of surgical patients. Also, this review identifies a gap in specialty-specific PREMs and a lack of psychometric validation for the promising PREMs. The prevalent use of generic PREMs, rather than PREMs specifically tailored to surgical patients, underscores the necessity for targeted measures to capture the unique experiences and priorities of surgical patients. Future research should prioritize the psychometric validation of PREMs utilized in surgical settings and developing and validating specialty-specific PREMs.

## Supplementary Material

zrae142_Supplementary_Data

## Data Availability

All data used in this systematic review are included in the article and its Supplementary material. Additional data and materials are available upon request from the corresponding author.
