# Supplementary material for: Surgical patient-reported experience measures and qualitative experience studies: systematic review
Source: BJS Open. 2025 Feb 11;9(1):zrae142. doi: 10.1093/bjsopen/zrae142 (PMC11811639; doi:10.1093/bjsopen/zrae142)
Supplement: zrae142_Supplementary_Data [file zrae142_supplementary_data.docx]

**Title *Surgical Patient Reported Experience Measures (PREMs) and Qualitative Experience Studies: systematic review***

**Authors** Maram Darwish^1,2^, Shabita Nandy^3^, Simone Willis^4,5^, James Coulson^3^, Kathleen Withers^4,6,7^, David Bosanquet^1,8^

**Affiliations** ^1^Southeast Wales Vascular Network, University Hospital of Wales, Cardiff, UK

^2^Health Education England, The East Midlands Deanery, Leicester, UK

^3^Cardiff University, School of Medicine, University Hospital of Wales, Heath Park, Cardiff, UK

^4^Centre for Healthcare Evaluation, Device Assessment and Research, CEDAR, Cardiff and Vale University Health Board, Cardiff, UK

^5^Specialist Unit for Review Evidence, Cardiff University, Cardiff, UK

^6^The Welsh Value in Health Centre, Cwm Taf University Health Board, UK

^7^Cardiff University, University Hospital of Wales, Heath Park, Cardiff, UK

^8^Department of Vascular Surgery, Aneurin Bevan University Health Board, UK

**Corresponding author** Mrs. Kathleen Withers

Cardiff University

Cardiff and Vale University Health Board Cardiff Medicentre, Heath Park

CF14 4UJ

Tel: +44 (0) 2920 744771

Email: [withersk@cardiff.ac.uk](mailto:withersk@cardiff.ac.uk)

ORCHID ID: [0000-0001-9514-2025](https://orcid.org/0000-0001-9514-2025)

**Supplementary Materials - Index**

| **Supplementary Methods** | |  |
| --- | --- | --- |
| PRISMA Checklist (supplementary S1) | | *page 3* |
| PRISMA-S Checklist (supplementary S2)  Deviations from the original protocol are depicted in supplementary S3  Database search strategy (Supplementary S4)  JBI critical appraisal checklist for case-control studies (Supplementary S5)  JBI critical appraisal checklist for cross sectional studies (Supplementary S6)  JBI critical appraisal checklist for qualitative studies (Supplementary S7). | *page 5*  *Page 6*  *Page 7*  *Page 26*  *Page 27*  *Page 28* | |
| **Supplementary Results** | |  |
| Excluded studies (Supplementary S8) | | *page 29* |
| PRISMA Flow diagram (Supplementary S9)  *Methodological quality of studies (Supplementary S10)*  Details of psychometric assessment in the translated versions of the GPNCS (Supplementary S11) | | *page 30*  *Page31*  *Page34* |
| **References** | | *Page 36* |
|  | |  |
|  | |  |
|  | |  |
|  | |  |
|  | |  |

**Supplementary Methods**

**Supplementary S1: PRISMA 2020 Checklist (1)**

| **Section and Topic** | **Item #** | **Checklist item** | **Location where item is reported** |
| --- | --- | --- | --- |
| **TITLE** | | |  |
| Title | 1 | Identify the report as a systematic review. | Page 1 |
| **ABSTRACT** | | |  |
| Abstract | 2 | See the PRISMA 2020 for Abstracts checklist. | Page 3 |
| **INTRODUCTION** | | |  |
| Rationale | 3 | Describe the rationale for the review in the context of existing knowledge. | Page 5 |
| Objectives | 4 | Provide an explicit statement of the objective(s) or question(s) the review addresses. | Page 6 |
| **METHODS** | | |  |
| Eligibility criteria | 5 | Specify the inclusion and exclusion criteria for the review and how studies were grouped for the syntheses. | Page 6,7 |
| Information sources | 6 | Specify all databases, registers, websites, organisations, reference lists and other sources searched or consulted to identify studies. Specify the date when each source was last searched or consulted. | Page 7 |
| Search strategy | 7 | Present the full search strategies for all databases, registers and websites, including any filters and limits used. | Page 7, and Supplementary S4 |
| Selection process | 8 | Specify the methods used to decide whether a study met the inclusion criteria of the review, including how many reviewers screened each record and each report retrieved, whether they worked independently, and if applicable, details of automation tools used in the process. | Page 7 |
| Data collection process | 9 | Specify the methods used to collect data from reports, including how many reviewers collected data from each report, whether they worked independently, any processes for obtaining or confirming data from study investigators, and if applicable, details of automation tools used in the process. | Page 8 |
| Data items | 10a | List and define all outcomes for which data were sought. Specify whether all results that were compatible with each outcome domain in each study were sought (e.g. for all measures, time points, analyses), and if not, the methods used to decide which results to collect. | Page 8, 9 |
|  | 10b | List and define all other variables for which data were sought (e.g. participant and intervention characteristics, funding sources). Describe any assumptions made about any missing or unclear information. | Page 8,9 |
| Study risk of bias assessment | 11 | Specify the methods used to assess risk of bias in the included studies, including details of the tool(s) used, how many reviewers assessed each study and whether they worked independently, and if applicable, details of automation tools used in the process. | Page 7,8 |
| Effect measures | 12 | Specify for each outcome the effect measure(s) (e.g. risk ratio, mean difference) used in the synthesis or presentation of results. | N/A |
| Synthesis methods | 13a | Describe the processes used to decide which studies were eligible for each synthesis (e.g. tabulating the study intervention characteristics and comparing against the planned groups for each synthesis (item #5)). | N/A |
|  | 13b | Describe any methods required to prepare the data for presentation or synthesis, such as handling of missing summary statistics, or data conversions. | N/A |
|  | 13c | Describe any methods used to tabulate or visually display results of individual studies and syntheses. | N/A |
|  | 13d | Describe any methods used to synthesize results and provide a rationale for the choice(s). If meta-analysis was performed, describe the model(s), method(s) to identify the presence and extent of statistical heterogeneity, and software package(s) used. | N/A |
|  | 13e | Describe any methods used to explore possible causes of heterogeneity among study results (e.g. subgroup analysis, meta-regression). | N/A |
|  | 13f | Describe any sensitivity analyses conducted to assess robustness of the synthesized results. | N/A |
| Reporting bias assessment | 14 | Describe any methods used to assess risk of bias due to missing results in a synthesis (arising from reporting biases). | N/A |
| Certainty assessment | 15 | Describe any methods used to assess certainty (or confidence) in the body of evidence for an outcome. | N/A |
| **RESULTS** | | |  |
| Study selection | 16a | Describe the results of the search and selection process, from the number of records identified in the search to the number of studies included in the review, ideally using a flow diagram. | Supplementary S9 |
|  | 16b | Cite studies that might appear to meet the inclusion criteria, but which were excluded, and explain why they were excluded. | Supplementary S8 |
| Study characteristics | 17 | Cite each included study and present its characteristics. | Table 1 (Supplementary S10), Table 2 |
| Risk of bias in studies | 18 | Present assessments of risk of bias for each included study. | Table 4,5,6 |
| Results of individual studies | 19 | For all outcomes, present, for each study: (a) summary statistics for each group (where appropriate) and (b) an effect estimate and its precision (e.g. confidence/credible interval), ideally using structured tables or plots. | N/A |
| Results of syntheses | 20a | For each synthesis, briefly summarise the characteristics and risk of bias among contributing studies. | Table 3 |
|  | 20b | Present results of all statistical syntheses conducted. If meta-analysis was done, present for each the summary estimate and its precision (e.g. confidence/credible interval) and measures of statistical heterogeneity. If comparing groups, describe the direction of the effect. | N/A |
|  | 20c | Present results of all investigations of possible causes of heterogeneity among study results. | N/A |
|  | 20d | Present results of all sensitivity analyses conducted to assess the robustness of the synthesized results. | N/A |
| Reporting biases | 21 | Present assessments of risk of bias due to missing results (arising from reporting biases) for each synthesis assessed. | N/A |
| Certainty of evidence | 22 | Present assessments of certainty (or confidence) in the body of evidence for each outcome assessed. | Table 3 |
| **DISCUSSION** | | |  |
| Discussion | 23a | Provide a general interpretation of the results in the context of other evidence. | Page 12,13,14, 15 |
|  | 23b | Discuss any limitations of the evidence included in the review. | Page 16 |
|  | 23c | Discuss any limitations of the review processes used. | Page 16 |
|  | 23d | Discuss implications of the results for practice, policy, and future research. | Page 16 |
| **OTHER INFORMATION** | | |  |
| Registration and protocol | 24a | Provide registration information for the review, including register name and registration number, or state that the review was not registered. | Page 6 |
|  | 24b | Indicate where the review protocol can be accessed, or state that a protocol was not prepared. | Page 6 |
|  | 24c | Describe and explain any amendments to information provided at registration or in the protocol. | Supplementary S3 |
| Support | 25 | Describe sources of financial or non-financial support for the review, and the role of the funders or sponsors in the review. | Page 2 |
| Competing interests | 26 | Declare any competing interests of review authors. | Page 2 |
| Availability of data, code and other materials | 27 | Report which of the following are publicly available and where they can be found: template data collection forms; data extracted from included studies; data used for all analyses; analytic code; any other materials used in the review. | Table 1 (S10),2,3,4,5,6  Supplementary: S1, S2, S3, S4, S5. S6, S7, S8, S9, S10, S11, S12 |

**Supplementary S2: PRISMA-S Checklist (2)**

**Supplementary S3: The deviation of the current manuscript from the original PROSPERO (3)**

The following points summarise the deviation of the current manuscript from the original PROSPERO protocol:

1. We included qualitative studies assessing surgical patients' experiences in addition to studies that used or developed PREMs as we believed they would add significant value to the review. However, there was no need to alter the inclusion/exclusion criteria, as it already encompassed 'studies assessing the patients' experiences in the peri-operative area,' which by default includes qualitative research.
2. We modified the title of our study from '*Assessing patients' experiences in surgery and surgical subspecialties: a systematic review of Patient Reported Experience Measures (PREMs*)' to *'*' ***A Systematic Review of surgical Patient Reported Experience Measures (PREMs) and Qualitative experience Studies’***. This alteration was made to incorporate qualitative studies that provide a deeper understanding of patient experiences, emotions, and perspectives. By including qualitative studies, we aim to enrich our assessment with nuanced insights that complement the quantitative data from PREMs, ultimately informing more patient-centred healthcare practices.
3. We have searched clinical trials registries including clinicaltrials.gov, EU Clinical Trials Register, and the ISRCTN registry using relevant terms. The search strategy included controlled vocabulary and free text terms encompassing the definition of PREMs, surgery, and surgical sub-specialties.

**Supplementary S4 (Database Search Strategy and Results)**

| Ovid MEDLINE(R) ALL <1946 to September 24, 2023> | | |
| --- | --- | --- |
| 1 | Patient satisfaction/ | 89633 |
| 2 | Personal satisfaction/ | 24442 |
| 3 | Patient Preference/ | 10719 |
| 4 | 1 or 2 or 3 | 122807 |
| 5 | outcome assessment (health care)/ | 82231 |
| 6 | treatment outcome/ | 1157018 |
| 7 | quality of life/ | 271285 |
| 8 | Activities of Daily Living/ | 73166 |
| 9 | Recovery of Function/ | 59243 |
| 10 | Postoperative Care/ | 60813 |
| 11 | Postoperative Complications/ | 401535 |
| 12 | Postoperative Pain/ | 48390 |
| 13 | exp Postoperative Period/ | 61837 |
| 14 | 5 or 6 or 7 or 8 or 9 or 10 or 11 or 12 or 13 | 1957978 |
| 15 | Surveys and Questionnaires/ | 564797 |
| 16 | exp health status indicators/ | 342848 |
| 17 | patient health questionnaire/ | 942 |
| 18 | Self Report/ | 43453 |
| 19 | 15 or 16 or 17 or 18 | 902208 |
| 20 | 4 and 14 and 19 | 14439 |
| 21 | experienc*.tw,kf. | 1423689 |
| 22 | 20 and 21 | 2821 |
| 23 | patient reported outcome measures/ and (experience* or satisf*).tw,kf. | 4229 |
| 24 | patient outcome assessment/ and (experience* or satisf*).tw,kf. | 1704 |
| 25 | ((patient* or client?) adj1 (perceived or perception* or important* or value*) adj1 (experienc* or satisf*)).tw,kf. | 239 |
| 26 | ((patient* or client?) adj1 (report* or measur* or questionnair* or scor$1 or scoring or assess* or survey* or interview*) adj1 experience*).tw,kf. | 1577 |
| 27 | (self adj1 (report* or measur* or questionnair* or scor$1 or scoring or assess* or survey* or interview*) adj1 (experience* or satisf*)).tw,kf. | 1171 |
| 28 | (PREM or PREMs or PREOM?).tw,kf. | 549 |
| 29 | 28 or 22 or 23 or 24 or 25 or 26 or 27 | 11437 |
| 30 | or/29 [PREMs] | 11437 |
| 31 | exp Specialties, Surgical/ | 220176 |
| 32 | exp Surgical Procedures, Operative/ | 3549823 |
| 33 | exp Surgeons/ | 17042 |
| 34 | su.fs. | 2262613 |
| 35 | (surger* or surgic* or surgeon* or procedure* or operation? or laparoscop* or postop*).tw,kf. | 3705485 |
| 36 | or/31-35 | 6095714 |
| 37 | 36 [PREMs Gen Surg] | 6095714 |
| 38 | exp vascular diseases/ | 1904508 |
| 39 | exp Digestive system diseases/ | 1961628 |
| 40 | exp Hernia/ | 83915 |
| 41 | exp Musculoskeletal Abnormalities/ or exp Musculoskeletal Diseases/ | 1212649 |
| 42 | exp Urologic Diseases/ or exp Male urogenital diseases/ or exp Female urogenital diseases/ or exp Prolapse/ | 1566250 |
| 43 | exp breast disease/ | 361454 |
| 44 | exp Neoplasms/ | 3867296 |
| 45 | exp Respiratory System diseases/ | 1730063 |
| 46 | exp Otorhinolaryngologic Diseases/ | 409897 |
| 47 | exp Eye Diseases/ | 638926 |
| 48 | exp Respiratory System Diseases/ | 1730063 |
| 49 | exp Hemorrhage/ | 365051 |
| 50 | exp Brain Diseases/ | 1410770 |
| 51 | (vascular or vascul* or arter* or arterial* or vein* or ven* or venous* or varicos* carotid* or aneurysm or aneurysm* or dissection or endarterectom* or subclavian or first rib or thoracic outlet* or sympathectom* or vascular access or fistula or aorta or aort* or ischemia or ischem* or femoral arter* or popliteal arter* or angioplast* or hemorrhage* or hematoma*).tw,kf. | 3384607 |
| 52 | exp Specialties, Surgical/ | 220176 |
| 53 | exp Surgical Procedures, Operative/ | 3549823 |
| 54 | su.fs. | 2262613 |
| 55 | (surger* or surgical* or operati* or reoperat* or bypass* or by-pass* or graft* or resect* or re-sect* or transplant* or procedure or procedures or debridement* or laparoscop* or laparotom*).tw,kf. | 4606594 |
| 56 | or/52-55 | 6610196 |
| 57 | 51 and 56 | 1296524 |
| 58 | or/41-51 | 11168075 |
| 59 | 57 and 58 | 1296524 |
| 60 | 59 [PREMs Surg condition] | 1296524 |
| 61 | ((opthalmolog* or eye* or vision or ocular or retina* or retinopath*) adj5 (operat* or procedur* or surger* or surgical*)).tw,kf. | 41602 |
| 62 | ((perforation* or incision* or laceration*) adj3 (repair* or drain* or closure*)).tw,kf. | 10667 |
| 63 | ((abdomen or abdominal or intestin* or bowel* or gastrointestin*) adj3 (ablat* or excis* or laparoscop* or laparotom* or operativ* or surger* or surgical* or reconstruct* or repair* or resect* or intraoperative* or perioperative* or perisurg* or postoperative* or postsurg* or preoperative* or presurg*)).tw,kf. | 82330 |
| 64 | (escharotom* or ((skin or derm*) adj2 (graft* or transplant*))).tw,kf. | 27931 |
| 65 | ((cancer or neoplas* or tumor* or tumour* or carcinom* or sarcoma*) adj3 (ablat* or excis* or laparoscop* or laparotom* or operativ* or surger* or surgical* or reconstruct* or repair* or resect* or biopsy or biopsie* or intraoperative* or perioperative* or perisurg* or postoperative* or postsurg* or preoperative* or presurg*)).tw,kf. | 245152 |
| 66 | (fundoplicat* or ((nissen* or toupet or dor) adj3 (operat* or procedur* or surger* or surgical*))).tw,kf. | 7164 |
| 67 | ((hernia* or extraperitoneal or preperitoneal or peritoneal or TEP or TAPP or umbilic* or inguinal or femoral or obturator) adj3 (ablat* or excis* or laparoscop* or laparotom* or operativ* or surger* or surgical* or reconstruct* or repair* or resect* or intraoperative* or perioperative* or perisurg* or postoperative* or postsurg* or preoperative* or presurg*)).tw,kf. | 40752 |
| 68 | ((liver or hepatic or lung or lungs or pulmon* or kidney) adj3 (transplant* or graft*)).tw,kf. | 159132 |
| 69 | (thoracoscop* or thoracotom* or pleurectom* or pleuroscop* or pleuracotom* or pleurotom* or (pleura* adj3 (endoscop* or incision*))).tw,kf. | 42818 |
| 70 | ((lung or lungs or pulmon* or wedge or trauma* or postrauma* or posttrauma* or neurotrauma* or fracture*) adj3 (ablat* or excis* or laparoscop* or laparotom* or operativ* or surger* or surgical* or reconstruct* or repair* or resect* or intraoperative* or perioperative* or perisurg* or postoperative* or postsurg* or preoperative* or presurg*)).tw,kf. | 119938 |
| 71 | ((breast or mastectom* or fibroadenoma*) adj3 (ablat* or excis* or operativ* or surger* or surgical* or reconstruct* or repair* or resect* or intraoperative* or perioperative* or perisurg* or postoperative* or postsurg* or preoperative* or presurg*)).tw,kf. | 41764 |
| 72 | (gastrostom or ileostom* or colostom* or enterostom* or portoenterostom or roux-en-y or whipple procedure or pancreatectom* or diverticulectom* or diverticulotom* or cholecystectom* or cholangiopancreatograph* or cholangio-pancreatograph* or choledoduodenostom* or choledo-duodenostom* or appendicectom* or appendectom* or splenectom* or pneumonectom* or amputation* or amputate* or craniotom* or craniostom* or hydrocelectom* or thoracostom* or fasciotom*).tw,kf. | 212247 |
| 73 | or/61-72 | 930623 |
| 74 | 30 and 51 and 73 [PREMs Surg spec] | 80 |
| 75 | Colorectal Surgery/ | 4428 |
| 76 | Rectal Diseases/ or exp *Rectal Diseases/ | 179566 |
| 77 | 75 or 76 | 182570 |
| 78 | 77 [PREMs Surg spec] | 182570 |
| 79 | 61 or 62 or 63 or 64 or 65 or 66 or 67 or 68 or 69 or 70 or 71 or 72 or 75 or 76 | 1085157 |
| 80 | 56 and 79 | 932406 |
| 81 | 29 and 35 and 79 | 727 |
| 82 | remove duplicates from 81 | 727 |

| PubMed ALL <1946 to August 24, 2023> | | | | | | |
| --- | --- | --- | --- | --- | --- | --- |
| 91 | | #22 AND #31 AND #77 AND #88 | | | | 2,194 |
| 89 | | #88 AND #77 | | | | 11,920 |
| 88 | | #86 OR #4 | | | | 123,455 |
| 87 | | #86 AND #77 | | | | 21 |
| 86 | | [PREMs] | | | | 297 |
| 85 | | "[PREMs Surgical]" | | | | 38 |
| 84 | | "[PREMs Surgical specialities]" | | | | 21 |
| 83 | | "[PREMs Surg spec]" | | | | 0 |
| 82 | | "[PREMs Surg spec]" - Schema: all | | | | 0 |
| 81 | | #80 OR #79 | | | | 204,679 |
| 80 | | "Rectal Diseases"[MeSH Terms:no exp] OR "Rectal Diseases"[MeSH Major Topic] | | | | 201,804 |
| 79 | | "Colorectal Surgery"[MeSH Terms:no exp] | | | | 4,448 |
| 78 | | #31 AND #63 AND #77 "[PREMs Surg spec]" | | | | 0 |
| 77 | | #76 OR #75 OR #74 OR #73 OR #72 OR #71 OR #70 OR #69 OR #68 OR #67 OR #66 OR #65 | | | | 2,112,201 |
| 76 | | (gastrostom[Title/Abstract] OR ileostom*[Title/Abstract] OR colostom*[Title/Abstract] OR enterostom*[Title/Abstract] OR portoenterostom[Title/Abstract] OR roux-en-y[Title/Abstract] OR "whipple procedure"[Title/Abstract] OR pancreatectom*[Title/Abstract] OR diverticulectom*[Title/Abstract] OR diverticulotom*[Title/Abstract] OR cholecystectom*[Title/Abstract] OR cholangiopancreatograph*[Title/Abstract] OR cholangio-pancreatograph*[Title/Abstract] OR choledoduodenostom*[Title/Abstract] OR choledo-duodenostom*[Title/Abstract] OR appendicectom*[Title/Abstract] OR appendectom*[Title/Abstract] OR splenectom*[Title/Abstract] OR pneumonectom*[Title/Abstract] OR amputation*[Title/Abstract] OR amputate*[Title/Abstract] OR craniotom*[Title/Abstract] OR craniostom*[Title/Abstract] OR hydrocelectom*[Title/Abstract] OR thoracostom*[Title/Abstract] OR fasciotom*[Title/Abstract]) | | | | 213,029 |
| 75 | | ((breast[Title/Abstract] OR mastectom*[Title/Abstract] OR fibroadenoma*[Title/Abstract]) AND (ablat*[Title/Abstract] OR excis*[Title/Abstract] OR operativ*[Title/Abstract] OR surger*[Title/Abstract] OR surgical*[Title/Abstract] OR reconstruct*[Title/Abstract] OR repair*[Title/Abstract] OR resect*[Title/Abstract] OR intraoperative*[Title/Abstract] OR perioperative*[Title/Abstract] OR perisurg*[Title/Abstract] OR postoperative*[Title/Abstract] OR postsurg*[Title/Abstract] OR preoperative*[Title/Abstract] OR presurg*[Title/Abstract])) | | | | 103,540 |
| 74 | | ((lung[Title/Abstract] OR lungs[Title/Abstract] OR pulmon*[Title/Abstract] OR wedge[Title/Abstract] OR trauma*[Title/Abstract] OR postrauma*[Title/Abstract] OR posttrauma*[Title/Abstract] OR neurotrauma*[Title/Abstract] OR fracture*[Title/Abstract]) AND (ablat*[Title/Abstract] OR excis*[Title/Abstract] OR laparoscop*[Title/Abstract] OR laparotom*[Title/Abstract] OR operativ*[Title/Abstract] OR surger*[Title/Abstract] OR surgical*[Title/Abstract] OR reconstruct*[Title/Abstract] OR repair*[Title/Abstract] OR resect*[Title/Abstract] OR intraoperative*[Title/Abstract] OR perioperative*[Title/Abstract] OR perisurg*[Title/Abstract] OR postoperative*[Title/Abstract] OR postsurg*[Title/Abstract] OR preoperative*[Title/Abstract] OR presurg*[Title/Abstract])) | | | | 462,793 |
| 73 | | (thoracoscop*[Title/Abstract] OR thoracotom*[Title/Abstract] OR pleurectom*[Title/Abstract] OR pleuroscop*[Title/Abstract] OR pleuracotom*[Title/Abstract] OR pleurotom*[Title/Abstract] OR (pleura*[Title/Abstract] AND (endoscop*[Title/Abstract] OR incision*[Title/Abstract]))) | | | | 44,267 |
| 72 | | ((liver[Title/Abstract] OR hepatic[Title/Abstract] OR lung[Title/Abstract] OR lungs[Title/Abstract] OR pulmon*[Title/Abstract] OR kidney[Title/Abstract]) AND (transplant*[Title/Abstract] OR graft*[Title/Abstract])) | | | | 220,585 |
| 71 | | ((hernia*[Title/Abstract] OR extraperitoneal[Title/Abstract] OR preperitoneal[Title/Abstract] OR peritoneal[Title/Abstract] OR TEP[Title/Abstract] OR TAPP[Title/Abstract] OR umbilic*[Title/Abstract] OR inguinal[Title/Abstract] OR femoral[Title/Abstract] OR obturator[Title/Abstract]) AND (ablat*[Title/Abstract] OR excis*[Title/Abstract] OR laparoscop*[Title/Abstract] OR laparotom*[Title/Abstract] OR operativ*[Title/Abstract] OR surger*[Title/Abstract] OR surgical*[Title/Abstract] OR reconstruct*[Title/Abstract] OR repair*[Title/Abstract] OR resect*[Title/Abstract] OR intraoperative*[Title/Abstract] OR perioperative*[Title/Abstract] OR perisurg*[Title/Abstract] OR postoperative*[Title/Abstract] OR postsurg*[Title/Abstract] OR preoperative*[Title/Abstract] OR presurg*[Title/Abstract])) | | | | 173,106 |
| 70 | | (fundoplicat*[Title/Abstract] OR ((nissen*[Title/Abstract] OR toupet[Title/Abstract] OR dor[Title/Abstract]) AND (operat*[Title/Abstract] OR procedur*[Title/Abstract] OR surger*[Title/Abstract] OR surgical*[Title/Abstract]))) | | | | 8,695 |
| 69 | | ((cancer[Title/Abstract] OR neoplas*[Title/Abstract] OR tumor*[Title/Abstract] OR tumour*[Title/Abstract] OR carcinom*[Title/Abstract] OR sarcoma*[Title/Abstract]) AND (ablat*[Title/Abstract] OR excis*[Title/Abstract] OR laparoscop*[Title/Abstract] OR laparotom*[Title/Abstract] OR operativ*[Title/Abstract] OR surger*[Title/Abstract] OR surgical*[Title/Abstract] OR reconstruct*[Title/Abstract] OR repair*[Title/Abstract] OR resect*[Title/Abstract] OR biopsy[Title/Abstract] OR biopsie*[Title/Abstract] OR intraoperative*[Title/Abstract] OR perioperative*[Title/Abstract] OR perisurg*[Title/Abstract] OR postoperative*[Title/Abstract] OR postsurg*[Title/Abstract] OR preoperative*[Title/Abstract] OR presurg*[Title/Abstract])) | | | | 964,186 |
| 68 | | (escharotom*[Title/Abstract] OR ((skin[Title/Abstract] OR derm*[Title/Abstract]) AND (graft*[Title/Abstract] OR transplant*[Title/Abstract]))) | | | | 52,521 |
| 67 | | ((abdomen[Title/Abstract] OR abdominal[Title/Abstract] OR intestin*[Title/Abstract] OR bowel*[Title/Abstract] OR gastrointestin*[Title/Abstract]) AND (ablat*[Title/Abstract] OR excis*[Title/Abstract] OR laparoscop*[Title/Abstract] OR laparotom*[Title/Abstract] OR operativ*[Title/Abstract] OR surger*[Title/Abstract] OR surgical*[Title/Abstract] OR reconstruct*[Title/Abstract] OR repair*[Title/Abstract] OR resect*[Title/Abstract] OR intraoperative*[Title/Abstract] OR perioperative*[Title/Abstract] OR perisurg*[Title/Abstract] OR postoperative*[Title/Abstract] OR postsurg*[Title/Abstract] OR preoperative*[Title/Abstract] OR presurg*[Title/Abstract])) | | | | 305,496 |
| 66 | | ((perforation*[Title/Abstract] OR incision*[Title/Abstract] OR laceration*[Title/Abstract]) AND (repair*[Title/Abstract] OR drain*[Title/Abstract] OR closure*[Title/Abstract])) | | | | 37,737 |
| 65 | | ((opthalmolog*[Title/Abstract] OR eye*[Title/Abstract] OR vision[Title/Abstract] OR ocular[Title/Abstract] OR retina*[Title/Abstract] OR retinopath*[Title/Abstract]) AND (operat*[Title/Abstract] OR procedur*[Title/Abstract] OR surger*[Title/Abstract] OR surgical*[Title/Abstract])) | | | | 118,290 |
| 64 | | "[PREMs Surg condition]" | | | | 3 |
| 63 | | #62 AND #60 | | | | 3,727,871 |
| 62 | | #40 OR #41 OR#42 OR #43 OR #44 OR #45 OR #46 OR #47 OR #48 OR #49 OR #50 OR #51 OR #52 OR #53 OR #54 OR #55 OR#56 OR #57 OR #58 | | | | ######## |
| 61 | | #58 AND #60 | | | | 1,127,932 |
| 60 | | #32 OR #33 OR #35 OR #59 | | | | 6,637,470 |
| 59 | | (surger*[Title/Abstract] OR surgical*[Title/Abstract] OR operati*[Title/Abstract] OR reoperat*[Title/Abstract] OR bypass*[Title/Abstract] OR by-pass*[Title/Abstract] OR graft*[Title/Abstract] OR resect*[Title/Abstract] OR re-sect*[Title/Abstract] OR transplant*[Title/Abstract] OR procedure[Title/Abstract] OR procedures[Title/Abstract] OR debridement*[Title/Abstract] OR laparoscop*[Title/Abstract] OR laparotom*[Title/Abstract]) | | | | 4,617,761 |
| 35 | | "Surgery"[Subheading] | | | | 2,337,969 |
| 33 | | "Surgical Procedures, Operative"[MeSH Terms] | | | | 3,555,368 |
| 32 | | "Specialties, Surgical"[MeSH Terms] | | | | 220,447 |
| 58 | | (vascular[Title/Abstract] OR vascul*[Title/Abstract] OR arter*[Title/Abstract] OR arterial*[Title/Abstract] OR vein*[Title/Abstract] OR ven*[Title/Abstract] OR venous*[Title/Abstract] OR "varicos* carotid*"[Title/Abstract] OR aneurysm[Title/Abstract] OR aneurysm*[Title/Abstract] OR dissection[Title/Abstract] OR endarterectom*[Title/Abstract] OR subclavian[Title/Abstract] OR "first rib"[Title/Abstract] OR "thoracic outlet*"[Title/Abstract] OR sympathectom*[Title/Abstract] OR "vascular access"[Title/Abstract] OR fistula[Title/Abstract] OR aorta[Title/Abstract] OR aort*[Title/Abstract] OR ischemia[Title/Abstract] OR ischem*[Title/Abstract] OR "femoral arter*"[Title/Abstract] OR "popliteal arter*"[Title/Abstract] OR angioplast*[Title/Abstract] OR hemorrhage*[Title/Abstract] OR hematoma*[Title/Abstract]) | | | | 2,704,345 |
| 57 | | "Brain Diseases"[MeSH Terms] | | | | 1,414,313 |
| 56 | | Hemorrhage[MeSH Terms] | | | | 365,675 |
| 55 | | "Respiratory System Diseases"[MeSH Terms] | | | | 0 |
| 54 | | "Respiratory System Diseases"[MeSH Terms] - Schema: all | | | | 0 |
| 53 | | "Eye Diseases"[MeSH Terms] | | | | 640,122 |
| 52 | | "Otorhinolaryngologic Diseases"[MeSH Terms] | | | | 410,550 |
| 51 | | "Respiratory System diseases"[MeSH Terms] | | | | 0 |
| 50 | | "Respiratory System diseases"[MeSH Terms] - Schema: all | | | | 0 |
| 49 | | Neoplasms[MeSH Terms] | | | | 3,876,003 |
| 48 | | "breast surgery"[MeSH Terms] | | | | 0 |
| 47 | | "breast surgery"[MeSH Terms] - Schema: all | | | | 0 |
| 46 | | "breast disease"[MeSH Terms] | | | | 0 |
| 45 | | "breast disease"[MeSH Terms] - Schema: all | | | | 0 |
| 44 | | "Urologic Diseases"[MeSH Terms] OR "Male urogenital diseases"[MeSH Terms] OR "Female urogenital diseases"[MeSH Terms] OR Prolapse[MeSH Terms] | | | | 1,568,801 |
| 43 | | "Musculoskeletal Abnormalities"[MeSH Terms] OR "Musculoskeletal Diseases"[MeSH Terms] | | | | 1,214,931 |
| 42 | | Hernia[MeSH Terms] | | | | 84,052 |
| 41 | | "Digestive system diseases"[MeSH Terms] | | | | 1,965,406 |
| 40 | | "vascular diseases"[MeSH Terms] | | | | 1,907,963 |
| 39 | | "[PREMs Gen Surg]" | | | | 0 |
| 38 | | "[PREMs Gen Surg]" - Schema: all | | | | 0 |
| 37 | | #32 OR #33 OR #34 OR #35 OR #36 | | | | 6,215,805 |
| 36 | | (surger*[Title/Abstract] OR surgic*[Title/Abstract] OR surgeon*[Title/Abstract] OR procedure*[Title/Abstract] OR operation*[Title/Abstract] OR laparoscop*[Title/Abstract] OR postop*[Title/Abstract]) | | | | 3,810,220 |
| 34 | | Surgeons[MeSH Terms] | | | | 17,198 |
| 31 | | #25 OR #26 OR #27 OR #28 OR #29 OR 30 | | | | 589,697 |
| 30 | | (PREM[Title/Abstract] OR PREMs[Title/Abstract] OR PREOM*[Title/Abstract]) | | | | 874 |
| 29 | | (self[Title/Abstract] AND (report*[Title/Abstract] OR measur*[Title/Abstract] OR questionnair*[Title/Abstract] OR scor*1[Title/Abstract] OR scoring[Title/Abstract] OR assess*[Title/Abstract] OR survey*[Title/Abstract] OR interview*[Title/Abstract]) AND (experience*[Title/Abstract] OR satisf*[Title/Abstract])) | | | | 126,263 |
| 28 | | ((patient*[Title/Abstract] OR client*[Title/Abstract]) AND (report*[Title/Abstract] OR measur*[Title/Abstract] OR questionnair*[Title/Abstract] OR scor*1[Title/Abstract] OR scoring[Title/Abstract] OR assess*[Title/Abstract] OR survey*[Title/Abstract] OR interview*[Title/Abstract]) AND experience*[Title/Abstract]) | | | | 408,110 |
| 27 | | ((patient*[Title/Abstract] OR client*[Title/Abstract]) AND (perceived[Title/Abstract] OR perception*[Title/Abstract] OR important*[Title/Abstract] OR value*[Title/Abstract]) AND (experienc*[Title/Abstract] OR satisf*[Title/Abstract])) | | | | 203,530 |
| 26 | | "patient outcome assessment"[MeSH Terms] AND (experience*[Title/Abstract] OR satisf*[Title/Abstract]) | | | | 6,225 |
| 25 | | "patient reported outcome measures"[MeSH Terms] AND (experience*[Title/Abstract] OR satisf*[Title/Abstract]) | | | | 4,283 |
| 24 | | #23 AND #22 | | | | 8,135 |
| 23 | | experienc*[Title/Abstract] | | | | 1,431,765 |
| 22 | | #21 AND #16 AND #4 | | | | 43,440 |
| 21 | | #15 OR #16 OR #17 OR #18 | | | | 3,155,943 |
| 20 | | "Self Report"[MeSH Terms] | | | | 43,588 |
| 19 | | "patient health questionnaire"[MeSH Terms] | | | | 950 |
| 18 | | "health status indicators"[MeSH Terms] | | | | 343,038 |
| 17 | | "Surveys and Questionnaires"[MeSH Terms] | | | | 1,216,771 |
| 16 | | #5 OR #6 OR #7 OR #8 OR #9 OR #10 OR #11 OR #12 OR #13 | | | | 2,124,634 |
| 15 | | "Postoperative Period"[MeSH Terms] | | | | 61,891 |
| 14 | | "Postoperative Pain"[MeSH Terms] | | | | 0 |
| 13 | | "Postoperative Pain"[MeSH Terms] - Schema: all | | | | 0 |
| 12 | | "Postoperative Complications"[MeSH Terms] | | | | 614,236 |
| 11 | | "Postoperative Care"[MeSH Terms] | | | | 60,811 |
| 10 | | "Recovery of Function"[MeSH Terms] | | | | 59,255 |
| 9 | | "Activities of Daily Living"[MeSH Terms] | | | | 120,953 |
| 8 | | "quality of life"[MeSH Terms] | | | | 272,932 |
| 7 | | "treatment outcome"[MeSH Terms] | | | | 1,251,564 |
| 6 | | "outcome assessment (health care)"[MeSH Terms] | | | | 0 |
| 5 | | "outcome assessment (health care)"[MeSH Terms] - Schema: all | | | | 0 |
| 4 | | #1 OR #2 OR #3 | | | | 123,202 |
| 3 | | "Patient Preference"[MeSH Terms:no exp] | | | | 10,738 |
| 2 | | "Personal satisfaction"[MeSH Terms:no exp] | | | | 24,767 |
| 1 | | "Patient satisfaction"[MeSH Terms:no exp] | | | | 99,773 |
| Embase <1996 to 2023 September 24> | | | | | | |
| 1 | Patient satisfaction/ | | | | 163411 | |
| 2 | Personal satisfaction/ | | | | 68017 | |
| 3 | Patient Preference/ | | | | 26436 | |
| 4 | or/1-3 | | | | 251719 | |
| 5 | outcome assessment/ | | | | 851128 | |
| 6 | treatment outcome/ | | | | 949783 | |
| 7 | quality of life/ | | | | 606982 | |
| 8 | daily life activity/ | | | | 106646 | |
| 9 | convalescence/ | | | | 56792 | |
| 10 | exp postoperative care/ | | | | 92785 | |
| 11 | postoperative complication/ | | | | 355850 | |
| 12 | postoperative pain/ | | | | 81741 | |
| 13 | or/5-12 | | | | 2718410 | |
| 14 | health care survey/ | | | | 24265 | |
| 15 | self report/ | | | | 151083 | |
| 16 | or/14-15 | | | | 174497 | |
| 17 | 4 and 13 and 16 and experienc*.tw,kf. | | | | 627 | |
| 18 | patient-reported outcome/ and (experience* or satisf*).tw,kf. | | | | 15238 | |
| 19 | ((patient* or client?) adj1 (perceived or perception* or important* or value*) adj1 (experienc* or satisf*)).tw,kf. | | | | 354 | |
| 20 | ((patient* or client?) adj1 (report* or measur* or questionnair* or scor$1 or scoring or assess* or survey* or interview*) adj1 experience*).tw,kf. | | | | 2452 | |
| 21 | (self adj1 (report* or measur* or questionnair* or scor$1 or scoring or assess* or survey* or interview*) adj1 (experience* or satisf*)).tw,kf. | | | | 1437 | |
| 22 | (PREM or PREMs or PREOM?).tw,kf. | | | | 930 | |
| 23 | or/17-22 | | | | 19962 | |
| 24 | exp surgery/ | | | | 5035694 | |
| 25 | exp surgeon/ | | | | 194921 | |
| 26 | (surger* or surgic* or surgeon* or procedure* or operation? or laparoscop* or postop*).tw,kf. | | | | 4098435 | |
| 27 | or/24-26 | | | | 6527317 | |
| 28 | exp digestive system disease/ | | | | 3170970 | |
| 29 | exp urogenital tract disease/ | | | | 2476497 | |
| 30 | exp hernia/ | | | | 113910 | |
| 31 | exp musculoskeletal system malformation/ or exp musculoskeletal disease/ | | | | 2257002 | |
| 32 | exp neoplasm/ | | | | 4663353 | |
| 33 | exp respiratory tract disease/ | | | | 2596150 | |
| 34 | exp ear nose throat disease/ | | | | 467111 | |
| 35 | exp eye disease/ | | | | 905665 | |
| 36 | exp vascular disease/ | | | | 2593973 | |
| 37 | exp breast disease/ | | | | 603840 | |
| 38 | exp Hemorrhage/ | | | | 1015254 | |
| 39 | exp Brain Diseases/ | | | | 2118276 | |
| 40 | (vascular or vascul* or arter* or arterial* or vein* or ven* or venous* or varicos* carotid* or aneurysm or aneurysm* or dissection or endarterectom* or subclavian or first rib or thoracic outlet* or sympathectom* or vascular access or fistula or aorta or aort* or ischemia or ischem* or femoral arter* or popliteal arter* or angioplast* or hemorrhage* or hematoma*).tw,kf. | | | | 3773547 | |
| 41 | or/28-40 | | | | 14483435 | |
| 42 | exp *surgery/ | | | | 2208079 | |
| 43 | su.fs. | | | | 2017777 | |
| 44 | (surger* or surgical* or operati* or reoperat* or bypass* or by-pass* or resect* or re-sect* or transplant* or procedure or procedures or debridement* or laparoscop* or laparotom*).tw,kf. | | | | 5056955 | |
| 45 | or/42-44 | | | | 6163604 | |
| 46 | 41 and 45 | | | | 4420174 | |
| 47 | ((opthalmolog* or eye* or vision or ocular or retina* or retinopath*) adj5 (operat* or procedur* or surger* or surgical*)).tw,kf. | | | | 44403 | |
| 48 | ((perforation* or incision* or laceration*) adj3 (repair* or drain* or closure*)).tw,kf. | | | | 13424 | |
| 49 | ((abdomen or abdominal or intestin* or bowel* or gastrointestin*) adj3 (ablat* or excis* or laparoscop* or laparotom* or operativ* or surger* or surgical* or reconstruct* or repair* or resect* or intraoperative* or perioperative* or perisurg* or postoperative* or postsurg* or preoperative* or presurg*)).tw,kf. | | | | 101496 | |
| 50 | (escharotom* or ((skin or derm*) adj2 (graft* or transplant*))).tw,kf. | | | | 22390 | |
| 51 | ((cancer or neoplas* or tumor* or tumour* or carcinom* or sarcoma*) adj3 (ablat* or excis* or laparoscop* or laparotom* or operativ* or surger* or surgical* or reconstruct* or repair* or resect* or biopsy or biopsie* or intraoperative* or perioperative* or perisurg* or postoperative* or postsurg* or preoperative* or presurg*)).tw,kf. | | | | 323856 | |
| 52 | (fundoplicat* or ((nissen* or toupet or dor) adj3 (operat* or procedur* or surger* or surgical*))).tw,kf. | | | | 10122 | |
| 53 | ((hernia* or extraperitoneal or preperitoneal or peritoneal or TEP or TAPP or umbilic* or inguinal or femoral or obturator) adj3 (ablat* or excis* or laparoscop* or laparotom* or operativ* or surger* or surgical* or reconstruct* or repair* or resect* or intraoperative* or perioperative* or perisurg* or postoperative* or postsurg* or preoperative* or presurg*)).tw,kf. | | | | 51565 | |
| 54 | ((liver or hepatic or lung or lungs or pulmon* or kidney) adj3 (transplant* or graft*)).tw,kf. | | | | 246147 | |
| 55 | (thoracoscop* or thoracotom* or pleurectom* or pleuroscop* or pleuracotom* or pleurotom* or (pleura* adj3 (endoscop* or incision*))).tw,kf. | | | | 50773 | |
| 56 | ((lung or lungs or pulmon* or wedge or trauma* or postrauma* or posttrauma* or neurotrauma* or fracture*) adj3 (ablat* or excis* or laparoscop* or laparotom* or operativ* or surger* or surgical* or reconstruct* or repair* or resect* or intraoperative* or perioperative* or perisurg* or postoperative* or postsurg* or preoperative* or presurg*)).tw,kf. | | | | 132423 | |
| 57 | ((breast or mastectom* or fibroadenoma*) adj3 (ablat* or excis* or operativ* or surger* or surgical* or reconstruct* or repair* or resect* or intraoperative* or perioperative* or perisurg* or postoperative* or postsurg* or preoperative* or presurg*)).tw,kf. | | | | 54278 | |
| 58 | (gastrostom or ileostom* or colostom* or enterostom* or portoenterostom or roux-en-y or whipple procedure or pancreatectom* or diverticulectom* or diverticulotom* or cholecystectom* or cholangiopancreatograph* or cholangio-pancreatograph* or choledoduodenostom* or choledo-duodenostom* or appendicectom* or appendectom* or splenectom* or pneumonectom* or amputation* or amputate* or craniotom* or craniostom* or hydrocelectom* or thoracostom* or fasciotom*).tw,kf. | | | | 241952 | |
| 59 | Colorectal Surgery/ | | | | 17707 | |
| 60 | Rectal Diseases/ or exp *Rectal Diseases/ | | | | 260451 | |
| 61 | or/47-60 | | | | 1381570 | |
| 62 | 41 or 45 or 47 or 48 or 49 or 50 or 51 or 52 or 53 or 54 or 55 or 56 or 57 or 58 or 59 or 60 | | | | 16243466 | |
| 63 | ((PREM or PREMs or PREOM? or (patient* or client?)) adj2 (report* or value* or important*) adj2 experienc*).ti,kf. | | | | 689 | |
| 64 | 62 and ((PREM or PREMs or PREOM? or (patient* or client?)) adj2 (report* or value* or important*) adj2 experienc*).ab. | | | | 2498 | |
| 65 | remove duplicates from 64 | | | | 2459 | |
| 66 | limit 65 to "remove medline records" | | | | 1365 | |
| CINAHL via EBSCOhost searched on Saturday, September 24, 2023 4:38:45 PM | | | | | | |
| S25 | | | S22 OR S23 OR S24 Limiters - Exclude MEDLINE records; Age Groups: All Adult | | 121 | |
| S24 | | | TI ( ((PREM or PREMs or PREOM?) and experienc*) ) OR AB ( ((PREM or PREMs or PREOM?) and experienc*) ) | | 134 | |
| S23 | | | (AB ((PREM or PREMs or PREOM?) or ((patient* or client?) N2 (report* or value* or important*) N2 experienc*)) AND (S6 OR S7 OR S8 OR S9 OR S10 OR S11 OR S12 OR S13 OR S14 OR S15 OR S16 OR S17 OR S18 OR S19 OR S20 OR S21) | | 510 | |
| S22 | | | TI ((PREM or PREMs or PREOM?) or ((patient* or client?) N2 (report* or value* or important*) N2 experienc*)) | | 358 | |
| S21 | | | TI ( ((breast or mastectom* or fibroadenoma*) N3 (ablat* or excis* or operativ* or surger* or surgical* or reconstruct* or repair* or resect* or intraoperative* or perioperative* or perisurg* or postoperative* or postsurg* or preoperative* or presurg*)) ) OR AB ( ((breast or mastectom* or fibroadenoma*) N3 (ablat* or excis* or operativ* or surger* or surgical* or reconstruct* or repair* or resect* or intraoperative* or perioperative* or perisurg* or postoperative* or postsurg* or preoperative* or presurg*)) ) | | 10,268 | |
| S20 | | | TI ( (surger* or surgic* or surgeon* or procedure* or operation? or laparoscop* or postop*) ) OR AB ( (surger* or surgic* or surgeon* or procedure* or operation? or laparoscop* or postop*) ) | | 714,993 | |
| S19 | | | TI ( (surger* or surgical* or operati* or reoperat* or bypass* or by-pass* or resect* or re-sect* or transplant* or procedure or procedures or debridement* or laparoscop* or laparotom*) ) OR AB ( (surger* or surgical* or operati* or reoperat* or bypass* or by-pass* or resect* or re-sect* or transplant* or procedure or procedures or debridement* or laparoscop* or laparotom*) ) | | 884,537 | |
| S18 | | | TI ( (vascular or vascul* or arter* or arterial* or vein* or ven* or venous* or varicos* carotid* or aneurysm or aneurysm* or dissection or endarterectom* or subclavian or first rib or thoracic outlet* or sympathectom* or vascular access or fistula or aorta or aort* or ischemia or ischem* or femoral arter* or popliteal arter* or angioplast* or hemorrhage* or hematoma*) ) OR AB ( (vascular or vascul* or arter* or arterial* or vein* or ven* or venous* or varicos* carotid* or aneurysm or aneurysm* or dissection or endarterectom* or subclavian or first rib or thoracic outlet* or sympathectom* or vascular access or fistula or aorta or aort* or ischemia or ischem* or femoral arter* or popliteal arter* or angioplast* or hemorrhage* or hematoma*) ) | | 605,852 | |
| S17 | | | TI ( ((lung or lungs or pulmon* or wedge or trauma* or postrauma* or posttrauma* or neurotrauma* or fracture*) N3 (ablat* or excis* or laparoscop* or laparotom* or operativ* or surger* or surgical* or reconstruct* or repair* or resect* or intraoperative* or perioperative* or perisurg* or postoperative* or postsurg* or preoperative* or presurg*)) ) OR AB ( ((lung or lungs or pulmon* or wedge or trauma* or postrauma* or posttrauma* or neurotrauma* or fracture*) N3 (ablat* or excis* or laparoscop* or laparotom* or operativ* or surger* or surgical* or reconstruct* or repair* or resect* or intraoperative* or perioperative* or perisurg* or postoperative* or postsurg* or preoperative* or presurg*)) ) | | 31,916 | |
| S16 | | | TI ( (thoracoscop* or thoracotom* or pleurectom* or pleuroscop* or pleuracotom* or pleurotom* or (pleura* N3 (endoscop* or incision*))) ) OR AB ( (thoracoscop* or thoracotom* or pleurectom* or pleuroscop* or pleuracotom* or pleurotom* or (pleura* N3 (endoscop* or incision*))) ) | | 6,674 | |
| S15 | | | TI ( ((liver or hepatic or lung or lungs or pulmon* or kidney) N3 (transplant* or graft*)) ) OR AB ( ((liver or hepatic or lung or lungs or pulmon* or kidney) N3 (transplant* or graft*)) ) | | 21,689 | |
| S14 | | | TI ( ((hernia* or extraperitoneal or preperitoneal or peritoneal or TEP or TAPP or umbilic* or inguinal or femoral or obturator) N3 (ablat* or excis* or laparoscop* or laparotom* or operativ* or surger* or surgical* or reconstruct* or repair* or resect* or intraoperative* or perioperative* or perisurg* or postoperative* or postsurg* or preoperative* or presurg*)) ) OR AB ( ((hernia* or extraperitoneal or preperitoneal or peritoneal or TEP or TAPP or umbilic* or inguinal or femoral or obturator) N3 (ablat* or excis* or laparoscop* or laparotom* or operativ* or surger* or surgical* or reconstruct* or repair* or resect* or intraoperative* or perioperative* or perisurg* or postoperative* or postsurg* or preoperative* or presurg*)) ) | | 12,019 | |
| S13 | | | TI ( (fundoplicat* or ((nissen* or toupet or dor) N3 (operat* or procedur* or surger* or surgical*))) ) OR AB ( (fundoplicat* or ((nissen* or toupet or dor) N3 (operat* or procedur* or surger* or surgical*))) ) | | 1,432 | |
| S12 | | | TI ( ((cancer or neoplas* or tumor* or tumour* or carcinom* or sarcoma*) N3 (ablat* or excis* or laparoscop* or laparotom* or operativ* or surger* or surgical* or reconstruct* or repair* or resect* or biopsy or biopsie* or intraoperative* or perioperative* or perisurg* or postoperative* or postsurg* or preoperative* or presurg*)) ) OR ( ((cancer or neoplas* or tumor* or tumour* or carcinom* or sarcoma*) N3 (ablat* or excis* or laparoscop* or laparotom* or operativ* or surger* or surgical* or reconstruct* or repair* or resect* or biopsy or biopsie* or intraoperative* or perioperative* or perisurg* or postoperative* or postsurg* or preoperative* or presurg*)) ) | | 115,188 | |
| S11 | | | TI ( (escharotom* or ((skin or derm*) N2 (graft* or transplant*))) ) OR AB ( (escharotom* or ((skin or derm*) N2 (graft* or transplant*))) ) | | 3,791 | |
| S10 | | | TI ( ((abdomen or abdominal or intestin* or bowel* or gastrointestin*) N3 (ablat* or excis* or laparoscop* or laparotom* or operativ* or surger* or surgical* or reconstruct* or repair* or resect* or intraoperative* or perioperative* or perisurg* or postoperative* or postsurg* or preoperative* or presurg*)) ) OR AB ( ((abdomen or abdominal or intestin* or bowel* or gastrointestin*) N3 (ablat* or excis* or laparoscop* or laparotom* or operativ* or surger* or surgical* or reconstruct* or repair* or resect* or intraoperative* or perioperative* or perisurg* or postoperative* or postsurg* or preoperative* or presurg*)) ) | | 17,495 | |
| S9 | | | TI ( ((perforation* or incision* or laceration*) N3 (repair* or drain* or closure*)) ) OR AB ( ((perforation* or incision* or laceration*) N3 (repair* or drain* or closure*)) ) | | 3,102 | |
| S8 | | | TI ( ((opthalmolog* or eye* or vision or ocular or retina* or retinopath*) N5 (operat* or procedur* or surger* or surgical*)) ) OR AB ( ((opthalmolog* or eye* or vision or ocular or retina* or retinopath*) N5 (operat* or procedur* or surger* or surgical*)) ) | | 6,141 | |
| S7 | | | TI ( (adenoidectom* or laryngectom* or laryngoplast* or laryngoscop* or pharygectom* or tonsillectom* or tympanoplast* or tracheostom* or tracheotom* or orchidopex* or orchiopex* or orchiectom* or orchidectom* or herniorrhaph* or hernioplast* or herniaplast* or "herni*-plast*" or herniotom* or circumcis* or gastrostom or ileostom* or colostom* or enterostom* or portoenterostom or "roux-en-y" or whipple* or or diverticulectom* or diverticulotom* or cholecystectom* or cholangiopancreatograph* or cholangio-pancreatograph* or choledoduodenostom* or choledo-duodenostom* or appendicectom* or appendectom* or splenectom* or pneumonectom* or amputation* or amputate* or craniotom* or craniostom* or hydrocelectom* or thoracostom* or fasciotom*) ) OR AB ( (adenoidectom* or laryngectom* or laryngoplast* or laryngoscop* or pharygectom* or tonsillectom* or tympanoplast* or tracheostom* or tracheotom* or orchidopex* or orchiopex* or orchiectom* or orchidectom* or herniorrhaph* or hernioplast* or herniaplast* or "herni*-plast*" or herniotom* or circumcis* or gastrostom or ileostom* or colostom* or enterostom* or portoenterostom or "roux-en-y" or whipple* or or diverticulectom* or diverticulotom* or cholecystectom* or cholangiopancreatograph* or cholangio-pancreatograph* or choledoduodenostom* or choledo-duodenostom* or appendicectom* or appendectom* or splenectom* or pneumonectom* or amputation* or amputate* or craniotom* or craniostom* or hydrocelectom* or thoracostom* or fasciotom*) ) | | 58,684 | |
| S6 | | | TI ( (surger* or surgic* or surgeon* or operati* or reoperat* or bypass* or by-pass* or resect* or re-sect* or transplant* or procedure* or debridement* or laparoscop* or laparotom* or postop*) ) OR AB ( (surger* or surgic* or surgeon* or operati* or reoperat* or bypass* or by-pass* or resect* or re-sect* or transplant* or procedure* or debridement* or laparoscop* or laparotom* or postop*) ) | | 930,982 | |
| S5 | | | S1 OR S2 OR S3 OR S4 | | 3,760 | |
| S4 | | | TI ( (PREM or PREMs or PREOM?) ) OR AB ( (PREM or PREMs or PREOM?) ) | | 202 | |
| S3 | | | TI ( ((self) N1 (report* or measur* or questionnair* or scor$1 or scoring or assess* or survey* or interview*) N1 (experience* or satisf*)) ) OR AB ( ((self or proxy*) N1 (report* or measur* or questionnair* or scor$1 or scoring or assess* or survey* or interview*) N1 (experience* or satisf*)) ) | | 1,420 | |
| S2 | | | TI ( ((patient* or client?) N1 (report* or measur* or questionnair* or scor$1 or scoring or assess* or survey* or interview*) N1 experience*) ) OR AB ( ((patient* or client?) N1 (report* or measur* or questionnair* or scor$1 or scoring or assess* or survey* or interview*) N1 experience*) ) | | 2,161 | |
| S1 | | | TI ( (patient* or client?) N1 (perceived or perception* or important* or value*) N1 (experienc* or satisf*)) ) OR AB ( (patient* or client?) N1 N1 (perceived or perception* or important* or value*) N1 (experienc* or satisf*))) ) | | 140 | |
| Cochrane Library  Date Run: 25/09/2023 03:02:59 | | | | | | |
| #1 | | | [mh ^"Patient satisfaction"] | 14789 | | |
| #2 | | | [mh ^"Personal satisfaction"] | 1326 | | |
| #3 | | | [mh ^"Patient Preference"] | 1217 | | |
| #4 | | | #1 OR #2 OR #3 | 17089 | | |
| #5 | | | [mh ^"outcome assessment (health care)"] | 9851 | | |
| #6 | | | [mh ^"treatment outcome"] | 171047 | | |
| #7 | | | [mh ^"quality of life"] | 43899 | | |
| #8 | | | [mh ^"Activities of Daily Living"] | 6073 | | |
| #9 | | | [mh ^"Recovery of Function"] | 6384 | | |
| #10 | | | [mh ^"Postoperative Care"] | 5480 | | |
| #11 | | | [mh ^"Postoperative Complications"] | 20757 | | |
| #12 | | | [mh ^"Postoperative Pain"] | 19114 | | |
| #13 | | | [mh "Postoperative Period"] | 8330 | | |
| #14 | | | #5 OR #6 OR #7 OR #8 OR #9 OR #10 OR #11 OR #12 OR #13 | 248581 | | |
| #15 | | | [mh ^"Surveys and Questionnaires"] | 32190 | | |
| #16 | | | [mh "health status indicators"] | 26956 | | |
| #17 | | | [mh ^"patient health questionnaire"] | 137 | | |
| #18 | | | [mh ^"Self Report"] | 4022 | | |
| #19 | | | #15 OR #16 OR #17 OR #18 | 59709 | | |
| #20 | | | #4 AND #14 AND #19 | 2181 | | |
| #21 | | | experienc*:ti,ab,kw | 140035 | | |
| #22 | | | #20 AND #21 | 315 | | |
| #23 | | | [mh ^"patient reported outcome measures"] AND (experience*:ti,ab,kw OR satisf*:ti,ab,kw) | 409 | | |
| #24 | | | [mh ^"patient outcome assessment"] AND (experience*:ti,ab,kw OR satisf*:ti,ab,kw) | 134 | | |
| #25 | | | ((patient*:ti,ab,kw OR client?:ti,ab,kw) NEAR/1 (perceived:ti,ab,kw OR perception*:ti,ab,kw OR important*:ti,ab,kw OR value*:ti,ab,kw) NEAR/1 (experienc*:ti,ab,kw OR satisf*:ti,ab,kw)) | 88 | | |
| #26 | | | ((patient*:ti,ab,kw OR client?:ti,ab,kw) NEAR/1 (report*:ti,ab,kw OR measur*:ti,ab,kw OR questionnair*:ti,ab,kw OR scor?1:ti,ab,kw OR scoring:ti,ab,kw OR assess*:ti,ab,kw OR survey*:ti,ab,kw OR interview*:ti,ab,kw) NEAR/1 experience*:ti,ab,kw) | 258 | | |
| #27 | | | (self:ti,ab,kw NEAR/1 (report*:ti,ab,kw OR measur*:ti,ab,kw OR questionnair*:ti,ab,kw OR scor?1:ti,ab,kw OR scoring:ti,ab,kw OR assess*:ti,ab,kw OR survey*:ti,ab,kw OR interview*:ti,ab,kw) NEAR/1 (experience*:ti,ab,kw OR satisf*:ti,ab,kw)) | 338 | | |
| #28 | | | (PREM:ti,ab,kw OR PREMs:ti,ab,kw OR PREOM?:ti,ab,kw) | 106 | | |
| #29 | | | #28 OR #22 OR #23 OR #24 OR #25 OR #26 OR #27 | 1584 | | |
| #30 | | | #29 NEXT PREMs | 4 | | |
| #31 | | | [mh "Specialties, Surgical"] | 3717 | | |
| #32 | | | [mh "Surgical Procedures, Operative"] | 166348 | | |
| #33 | | | [mh Surgeons] | 341 | | |
| #34 | | | [mh /SU] | 79954 | | |
| #35 | | | (surger*:ti,ab,kw OR surgic*:ti,ab,kw OR surgeon*:ti,ab,kw OR procedure*:ti,ab,kw OR operation?:ti,ab,kw OR laparoscop*:ti,ab,kw OR postop*:ti,ab,kw) | 557241 | | |
| #36 | | | #31 OR #32 OR #33 OR #34 OR #35 | 598835 | | |
| #37 | | | "[PREMs Gen Surg]" | 0 | | |
| #38 | | | [mh "vascular diseases"] | 113839 | | |
| #39 | | | [mh "Digestive system diseases"] | 75244 | | |
| #40 | | | [mh Hernia] | 4039 | | |
| #41 | | | [mh "Musculoskeletal Abnormalities"] OR [mh "Musculoskeletal Diseases"] | 57509 | | |
| #42 | | | [mh "Urologic Diseases"] OR [mh "Male urogenital diseases"] OR [mh "Female urogenital diseases"] OR [mh Prolapse] | 67791 | | |
| #43 | | | [mh "breast disease"] | 18498 | | |
| #44 | | | [mh Neoplasms] | 112562 | | |
| #45 | | | [mh "Respiratory System diseases"] | 85896 | | |
| #46 | | | [mh "Otorhinolaryngologic Diseases"] | 17601 | | |
| #47 | | | [mh "Eye Diseases"] | 25588 | | |
| #48 | | | [mh "Respiratory System Diseases"] | 85896 | | |
| #49 | | | [mh Hemorrhage] | 19886 | | |
| #50 | | | [mh "Brain Diseases"] | 58318 | | |
| #51 | | | (vascular:ti,ab,kw OR vascul*:ti,ab,kw OR arter*:ti,ab,kw OR arterial*:ti,ab,kw OR vein*:ti,ab,kw OR ven*:ti,ab,kw OR venous*:ti,ab,kw OR (varicos* NEXT carotid*):ti,ab,kw OR aneurysm:ti,ab,kw OR aneurysm*:ti,ab,kw OR dissection:ti,ab,kw OR endarterectom*:ti,ab,kw OR subclavian:ti,ab,kw OR "first rib":ti,ab,kw OR ("thoracic" NEXT outlet*):ti,ab,kw OR sympathectom*:ti,ab,kw OR "vascular access":ti,ab,kw OR fistula:ti,ab,kw OR aorta:ti,ab,kw OR aort*:ti,ab,kw OR ischemia:ti,ab,kw OR ischem*:ti,ab,kw OR ("femoral" NEXT arter*):ti,ab,kw OR ("popliteal" NEXT arter*):ti,ab,kw OR angioplast*:ti,ab,kw OR hemorrhage*:ti,ab,kw OR hematoma*:ti,ab,kw) | 319276 | | |
| #52 | | | [mh "Specialties, Surgical"] | 3717 | | |
| #53 | | | [mh "Surgical Procedures, Operative"] | 166348 | | |
| #54 | | | [mh /SU] | 79954 | | |
| #55 | | | (surger*:ti,ab,kw OR surgical*:ti,ab,kw OR operati*:ti,ab,kw OR reoperat*:ti,ab,kw OR bypass*:ti,ab,kw OR by-pass*:ti,ab,kw OR graft*:ti,ab,kw OR resect*:ti,ab,kw OR re-sect*:ti,ab,kw OR transplant*:ti,ab,kw OR procedure:ti,ab,kw OR procedures:ti,ab,kw OR debridement*:ti,ab,kw OR laparoscop*:ti,ab,kw OR laparotom*:ti,ab,kw) | 592259 | | |
| #56 | | | #52 OR #53 OR #54 OR #55 | 626727 | | |
| #57 | | | #51 AND #56 | 150076 | | |
| #58 | | | #41 OR #42 OR #43 OR #44 OR #45 OR #46 OR #47 OR #48 OR #49 OR #50 OR #51 | 624448 | | |
| #59 | | | #57 AND #58 | 150076 | | |
| #60 | | | "[PREMs Surg condition]" | 0 | | |
| #61 | | | ((opthalmolog*:ti,ab,kw OR eye*:ti,ab,kw OR vision:ti,ab,kw OR ocular:ti,ab,kw OR retina*:ti,ab,kw OR retinopath*:ti,ab,kw) NEAR/5 (operat*:ti,ab,kw OR procedur*:ti,ab,kw OR surger*:ti,ab,kw OR surgical*:ti,ab,kw)) | 9038 | | |
| #62 | | | ((perforation*:ti,ab,kw OR incision*:ti,ab,kw OR laceration*:ti,ab,kw) NEAR/3 (repair*:ti,ab,kw OR drain*:ti,ab,kw OR closure*:ti,ab,kw)) | 1820 | | |
| #63 | | | ((abdomen:ti,ab,kw OR abdominal:ti,ab,kw OR intestin*:ti,ab,kw OR bowel*:ti,ab,kw OR gastrointestin*:ti,ab,kw) NEAR/3 (ablat*:ti,ab,kw OR excis*:ti,ab,kw OR laparoscop*:ti,ab,kw OR laparotom*:ti,ab,kw OR operativ*:ti,ab,kw OR surger*:ti,ab,kw OR surgical*:ti,ab,kw OR reconstruct*:ti,ab,kw OR repair*:ti,ab,kw OR resect*:ti,ab,kw OR intraoperative*:ti,ab,kw OR perioperative*:ti,ab,kw OR perisurg*:ti,ab,kw OR postoperative*:ti,ab,kw OR postsurg*:ti,ab,kw OR preoperative*:ti,ab,kw OR presurg*:ti,ab,kw)) | 17755 | | |
| #64 | | | (escharotom*:ti,ab,kw OR ((skin:ti,ab,kw OR derm*:ti,ab,kw) NEAR/2 (graft*:ti,ab,kw OR transplant*:ti,ab,kw))) | 1895 | | |
| #65 | | | ((cancer:ti,ab,kw OR neoplas*:ti,ab,kw OR tumor*:ti,ab,kw OR tumour*:ti,ab,kw OR carcinom*:ti,ab,kw OR sarcoma*:ti,ab,kw) NEAR/3 (ablat*:ti,ab,kw OR excis*:ti,ab,kw OR laparoscop*:ti,ab,kw OR laparotom*:ti,ab,kw OR operativ*:ti,ab,kw OR surger*:ti,ab,kw OR surgical*:ti,ab,kw OR reconstruct*:ti,ab,kw OR repair*:ti,ab,kw OR resect*:ti,ab,kw OR biopsy:ti,ab,kw OR biopsie*:ti,ab,kw OR intraoperative*:ti,ab,kw OR perioperative*:ti,ab,kw OR perisurg*:ti,ab,kw OR postoperative*:ti,ab,kw OR postsurg*:ti,ab,kw OR preoperative*:ti,ab,kw OR presurg*:ti,ab,kw)) | 46358 | | |
| #66 | | | (fundoplicat*:ti,ab,kw OR ((nissen*:ti,ab,kw OR toupet:ti,ab,kw OR dor:ti,ab,kw) NEAR/3 (operat*:ti,ab,kw OR procedur*:ti,ab,kw OR surger*:ti,ab,kw OR surgical*:ti,ab,kw))) | 810 | | |
| #67 | | | ((hernia*:ti,ab,kw OR extraperitoneal:ti,ab,kw OR preperitoneal:ti,ab,kw OR peritoneal:ti,ab,kw OR TEP:ti,ab,kw OR TAPP:ti,ab,kw OR umbilic*:ti,ab,kw OR inguinal:ti,ab,kw OR femoral:ti,ab,kw OR obturator:ti,ab,kw) NEAR/3 (ablat*:ti,ab,kw OR excis*:ti,ab,kw OR laparoscop*:ti,ab,kw OR laparotom*:ti,ab,kw OR operativ*:ti,ab,kw OR surger*:ti,ab,kw OR surgical*:ti,ab,kw OR reconstruct*:ti,ab,kw OR repair*:ti,ab,kw OR resect*:ti,ab,kw OR intraoperative*:ti,ab,kw OR perioperative*:ti,ab,kw OR perisurg*:ti,ab,kw OR postoperative*:ti,ab,kw OR postsurg*:ti,ab,kw OR preoperative*:ti,ab,kw OR presurg*:ti,ab,kw)) | 8361 | | |
| #68 | | | ((liver:ti,ab,kw OR hepatic:ti,ab,kw OR lung:ti,ab,kw OR lungs:ti,ab,kw OR pulmon*:ti,ab,kw OR kidney:ti,ab,kw) NEAR/3 (transplant*:ti,ab,kw OR graft*:ti,ab,kw)) | 16080 | | |
| #69 | | | (thoracoscop*:ti,ab,kw OR thoracotom*:ti,ab,kw OR pleurectom*:ti,ab,kw OR pleuroscop*:ti,ab,kw OR pleuracotom*:ti,ab,kw OR pleurotom*:ti,ab,kw OR (pleura*:ti,ab,kw NEAR/3 (endoscop*:ti,ab,kw OR incision*:ti,ab,kw))) | 4769 | | |
| #70 | | | ((lung:ti,ab,kw OR lungs:ti,ab,kw OR pulmon*:ti,ab,kw OR wedge:ti,ab,kw OR trauma*:ti,ab,kw OR postrauma*:ti,ab,kw OR posttrauma*:ti,ab,kw OR neurotrauma*:ti,ab,kw OR fracture*:ti,ab,kw) NEAR/3 (ablat*:ti,ab,kw OR excis*:ti,ab,kw OR laparoscop*:ti,ab,kw OR laparotom*:ti,ab,kw OR operativ*:ti,ab,kw OR surger*:ti,ab,kw OR surgical*:ti,ab,kw OR reconstruct*:ti,ab,kw OR repair*:ti,ab,kw OR resect*:ti,ab,kw OR intraoperative*:ti,ab,kw OR perioperative*:ti,ab,kw OR perisurg*:ti,ab,kw OR postoperative*:ti,ab,kw OR postsurg*:ti,ab,kw OR preoperative*:ti,ab,kw OR presurg*:ti,ab,kw)) | 18278 | | |
| #71 | | | ((breast:ti,ab,kw OR mastectom*:ti,ab,kw OR fibroadenoma*:ti,ab,kw) NEAR/3 (ablat*:ti,ab,kw OR excis*:ti,ab,kw OR operativ*:ti,ab,kw OR surger*:ti,ab,kw OR surgical*:ti,ab,kw OR reconstruct*:ti,ab,kw OR repair*:ti,ab,kw OR resect*:ti,ab,kw OR intraoperative*:ti,ab,kw OR perioperative*:ti,ab,kw OR perisurg*:ti,ab,kw OR postoperative*:ti,ab,kw OR postsurg*:ti,ab,kw OR preoperative*:ti,ab,kw OR presurg*:ti,ab,kw)) | 8304 | | |
| #72 | | | (gastrostom:ti,ab,kw OR ileostom*:ti,ab,kw OR colostom*:ti,ab,kw OR enterostom*:ti,ab,kw OR portoenterostom:ti,ab,kw OR roux-en-y:ti,ab,kw OR "whipple procedure":ti,ab,kw OR pancreatectom*:ti,ab,kw OR diverticulectom*:ti,ab,kw OR diverticulotom*:ti,ab,kw OR cholecystectom*:ti,ab,kw OR cholangiopancreatograph*:ti,ab,kw OR cholangio-pancreatograph*:ti,ab,kw OR choledoduodenostom*:ti,ab,kw OR choledo-duodenostom*:ti,ab,kw OR appendicectom*:ti,ab,kw OR appendectom*:ti,ab,kw OR splenectom*:ti,ab,kw OR pneumonectom*:ti,ab,kw OR amputation*:ti,ab,kw OR amputate*:ti,ab,kw OR craniotom*:ti,ab,kw OR craniostom*:ti,ab,kw OR hydrocelectom*:ti,ab,kw OR thoracostom*:ti,ab,kw OR fasciotom*:ti,ab,kw) | 22421 | | |
| #73 | | | #61 OR #62 OR #63 OR #64 OR #65 OR #66 OR #67 OR #68 OR #69 OR #70 OR #71 OR #72 | 133121 | | |
| #74 | | | #30 AND #51 AND #73 "[PREMs Surg spec]" | 0 | | |
| #75 | | | [mh ^"Colorectal Surgery"] | 428 | | |
| #76 | | | [mh ^"Rectal Diseases"] OR [mh "Rectal Diseases"] | 11900 | | |
| #77 | | | #75 OR #76 | 12245 | | |
| #78 | | | "[PREMs Surg spec]" | 0 | | |
| #79 | | | #61 OR #62 OR #63 OR #64 OR #65 OR #66 OR #67 OR #68 OR #69 OR #70 OR #71 OR #72 OR #75 OR #76 | 141872 | | |
| #80 | | | #56 AND #79 | 129827 | | |
| #81 | | | #29 AND #35 AND #79 | 108 | | |

Supplementary S5: JBI Critical appraisal checklist for case control studies(4)

**Supplementary S6:** JBI Critical appraisal checklist for analytical cross-sectional studies**** (4)

**Supplementary S7:** JBI Critical appraisal checklist for qualitative studies (4)

**Supplementary Results**

**Supplementary S8: Excluded Studies**

**
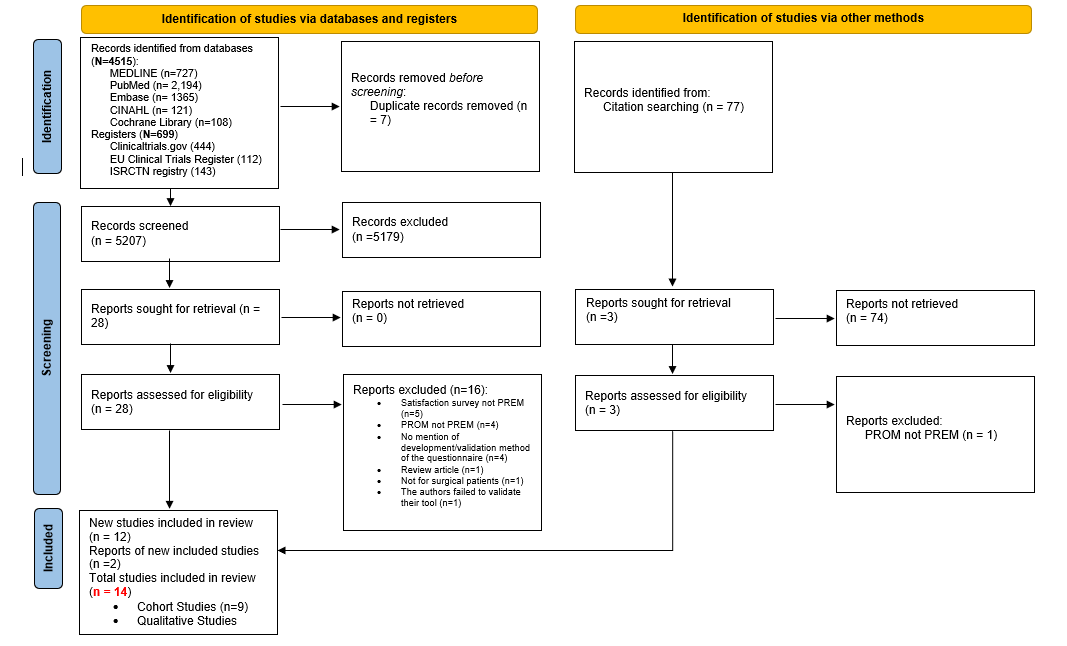
Supplementary document S9. PRISMA 2020 flow diagram for new systematic reviews which included searches of databases, registers and other sources (5)**

| **JBI Critical appraisal checklist for case-control studies reviewed** | |
| --- | --- |
| **Criteria** | **Schreiter et al, 2021** |
| 1.Were the groups comparable other than the presence of disease in cases or the absence of disease in controls? |  |
| 2.Were cases and controls matched appropriately? |  |
| 3.Were the same criteria used for identification of cases and controls? |  |
| 4.Was exposure measured in a standard, valid and reliable way? |  |
| 5.Was exposure measured in the same way for cases and controls? |  |
| 6.Were confounding factors identified? |  |
| 7.Were strategies to deal with confounding factors stated? |  |
| 8.Were outcomes assessed in a standard, valid and reliable way for cases and controls? |  |
| 9.Was the exposure period of interest long enough to be meaningful? |  |
| 10.Was appropriate statistical analysis used? |  |
| The red color means “No”, the green “Yes”, the yellow “Can not tell according to the JBI predefined criteria. | |

***Methodological quality of studies (Supplementary S10)***

| **JBI Critical appraisal checklist for analytical cross-sectional studies reviewed** | | | | | | | | |
| --- | --- | --- | --- | --- | --- | --- | --- | --- |
| **Criteria** | **Wick et al, 2015** | **Jones et al, 2017** | **Liu et al, 2018** | **Hertel-Joergensen et al, 2018** | **Donmez and Ozbayır, 2010** | **Murphy et al, 2018** | **Poelstra et al, 2018** | **Black et al, 2014** |
| 1.Were the criteria for inclusion in the sample clearly defined? |  |  |  |  |  |  |  |  |
| 2.Were the study subjects and the setting described in detail? |  |  |  |  |  |  |  |  |
| 3.Was the exposure measured in a valid and reliable way? |  |  |  |  |  |  |  |  |
| 4.Were objective, standard criteria used for measurement of the condition? |  |  |  |  |  |  |  |  |
| 5.Were confounding factors identified? |  |  |  |  |  |  |  |  |
| 6.Were strategies to deal with confounding factors stated? |  |  |  |  |  |  |  |  |
| 7.Were the outcomes measured in a valid and reliable way? |  |  |  |  |  |  |  |  |
| 8.Was appropriate statistical analysis used? |  |  |  |  |  |  |  |  |
| The red color means “No”, the green “Yes”, the yellow “Not applicable” according to the JBI predefined criteria. | | | | | | | | |

| **JBI Critical appraisal checklist for qualitative studies reviewed** | | | | | |
| --- | --- | --- | --- | --- | --- |
| **Criteria** | **Ventura-Aguiar et al, 2022** | **Harrison et al, 2023** | **Brennan et al, 2020** | **Arvidsson et al, 2023** | **Lithner et al, 2015** |
| 1. Is there congruity between the stated philosophical perspective and the research methodology? |  |  |  |  |  |
| 2.Is there congruity between the research methodology and the research question or objectives? |  |  |  |  |  |
| 3.Is there congruity between the research methodology and the methods used to collect data? |  |  |  |  |  |
| 4.Is there congruity between the research methodology and the representation and analysis of data? |  |  |  |  |  |
| 5.Is there congruity between the research methodology and the interpretation of results? |  |  |  |  |  |
| 6.Is there a statement locating the researcher culturally or theoretically? |  |  |  |  |  |
| 7.Is the influence of the researcher on the research, and vice- versa, addressed? |  |  |  |  |  |
| 8.Are participants, and their voices, adequately represented? |  |  |  |  |  |
| 9.Is the research ethical according to current criteria or, for recent studies, and is there evidence of ethical approval by an appropriate body? |  |  |  |  |  |
| 10.Do the conclusions drawn in the research report flow from the analysis, or interpretation, of the data? |  |  |  |  |  |
| The red color means “No”, the green “Yes”, the yellow “Not applicable” according to the JBI predefined criteria. | | | | | |

**Supplementary S11: Additional details of psychometric assessment in the translated versions of the GPNCS**

*structural validity*

The assessment of the structural validity for the Turkish translated version of GPNCS (6) was done according to the following:

Seven factors were obtained in the basic components analysis of the 32-item GPNCS, each factor explained a variance between 3.555–35.123% for a total of 68.939% of the variance explained. As a result of confirmatory factor analysis, the factor constructs were found to be consistent with the original scale. In calculating factors, the Eigenvalues were taken into consideration. Every factor’s eigenvalue was also found to be >1. The Kaiser–Meyer–Olkin (KMO) test was performed for the determination of sample size, the KMO coefficient was found to be 0.81 (excellent) and the Barlett test result was found to be highly significant (v2 = 8294.72, p < 0.001).

In the Danish version(7), the authors conducted a confirmatory factor analysis (CFA) for all the scale factors and calculated the model fit, the model fit estimates (CFI =0.73, TLI =0.70, RMSEA =0.095 [90% CI: 0.09-0.10]) and likelihood ratio = 1484(506).

*Reliability assessment*

In the Turkish version of GPNCS (6), the authors used Cronbach alpha and the split half test to assess the reliability, the correlation value between the two halves of the scale was 0.80, the Cronbach’s alpha coefficient was 0.83 for the first half (16 items) and 0.91 for the second half (16 items) with a Spearman-Brown coefficient of 0.88 and a Guttman Split Half Reliability coefficient of 0.88 which indicate a high level of reliability.

In the Danish version(7), Cronbach alpha values were calculated for each factor and for the total scale. Coefficients ranged from 0.72 to 0.85, with the total scale coefficient being 0.92.

**References**

1. Page MJ, Moher D, Bossuyt PM, Boutron I, Hoffmann TC, Mulrow CD, et al. PRISMA 2020 explanation and elaboration: updated guidance and exemplars for reporting systematic reviews. bmj. 2021;372.

2. Rethlefsen ML, Kirtley S, Waffenschmidt S, Ayala AP, Moher D, Page MJ, et al. PRISMA-S: an extension to the PRISMA statement for reporting literature searches in systematic reviews. Systematic reviews. 2021;10:1-19.

3. Darwish M, Withers K, Bosanquet D. Assessing patients' experiences in surgery and surgical subspecialties: a systematic review of Patient Reported Experience Measures (PREMs). PROSPERO 2023 CRD42023479711. 2023.

4. Moola S, Munn Z, Tufanaru C, Aromataris E, Sears K, Sfetcu R, et al. Systematic reviews of etiology and risk. Joanna Briggs Institute reviewer’s manual. 5: The Joanna Briggs Institute Adelaide, Australia; 2017. p. 217-69.

5. Parums DV. Review articles, systematic reviews, meta-analysis, and the updated preferred reporting items for systematic reviews and meta-analyses (PRISMA) 2020 guidelines. Medical science monitor: international medical journal of experimental and clinical research. 2021;27:e934475-1.

6. Donmez YC, Ozbayır T. Validity and reliability of the ‘good perioperative nursing care scale’for Turkish patients and nurses. Journal of Clinical Nursing. 2011;20(1‐2):166-74.

7. Hertel-Joergensen M, Abrahamsen C, Jensen C. Translation, adaptation and psychometric validation of the Good Perioperative Nursing Care Scale (GPNCS) with surgical patients in perioperative care. International journal of orthopaedic and trauma nursing. 2018;29:41-8.
